# Genome assembly, comparative genomics, and identification of genes/pathways underlying plant growth-promoting traits of an actinobacterial strain, *Amycolatopsis* sp. (BCA-696)

**DOI:** 10.1038/s41598-024-66835-y

**Published:** 2024-07-10

**Authors:** Prasad Gandham, Nandini Vadla, Angeo Saji, Vadlamudi Srinivas, Pradeep Ruperao, Sivasubramani Selvanayagam, Rachit K. Saxena, Abhishek Rathore, Subramaniam Gopalakrishnan, Vivek Thakur

**Affiliations:** 1 International Crops Research Institute for the Semi-Arid Tropics (ICRISAT), Hyderabad, India; 2 Department of Systems and Computational Biology, School of Life Sciences, University of Hyderabad, Hyderabad, India; 3 Gujarat Biotechnology University, Gandhinagar, Gujarat India; 4 Excellence in Breeding, International Maize and Wheat Improvement Center (CIMMYT), Hyderabad, India; 5 School of Plant, Environmental and Soil Sciences, Louisiana State University Agricultural Center, Baton Rouge, LA USA; 6 International Institute of Tropical Agriculture (IITA), Dar es Salaam, Tanzania

**Keywords:** Bacterial genomics, Rhizobial symbiosis, Plant immunity, Antifungal agents

## Abstract

The draft genome sequence of an agriculturally important actinobacterial species *Amycolatopsis* sp. BCA-696 was developed and characterized in this study. *Amycolatopsis* BCA-696 is known for its biocontrol properties against charcoal rot and also for plant growth-promotion (PGP) in several crop species. The next-generation sequencing (NGS)-based draft genome of *Amycolatopsis* sp. BCA-696 comprised of ~ 9.05 Mb linear chromosome with 68.75% GC content. In total, 8716 protein-coding sequences and 61 RNA-coding sequences were predicted in the genome. This newly developed genome sequence has been also characterized for biosynthetic gene clusters (BGCs) and biosynthetic pathways. Furthermore, we have also reported that the *Amycolatopsis* sp. BCA-696 produces the glycopeptide antibiotic vancomycin that inhibits the growth of pathogenic gram-positive bacteria. A comparative analysis of the BCA-696 genome with publicly available closely related genomes of 14 strains of *Amycolatopsis* has also been conducted. The comparative analysis has identified a total of 4733 core and 466 unique orthologous genes present in the BCA-696 genome The unique genes present in BCA-696 was enriched with antibiotic biosynthesis and resistance functions. Genome assembly of the BCA-696 has also provided genes involved in key pathways related to PGP and biocontrol traits such as siderophores, chitinase, and cellulase production.

## Introduction

The relationships of microbes with plants have been of diverse types, among which plant growth promotion (PGP) activities of microbes is one of the beneficial types to the plants. Beyond the lab or field-level characterizations of the PGP traits, there have been several recent studies where genomic approaches were used for the characterization of plant growth-promoting traits in rhizosphere bacteria. Some of the PGP traits studied include siderophores and indole acetic acid (IAA) production, anti-fungal properties, mineral solubilization, nitrogen fixation, activity of enzymes such as ACC deaminase, lipase, chitinase and cellulase^1–13^. Our group also reported draft genomes of sixteen PGP strains of *Streptomyces* and predicted genes underlying important PGP or biocontrol traits^14^. A typical approach used by these studies involves the decoding of genome sequence followed by identification of unique genomic islands, prediction of Biosynthetic Gene Clusters (BGC), comparison of BGCs as well as metabolic pathways across genomes, etc.

*Amycolatopsis* sp. BCA-696, one of the strains of an important genus of actinobacteria, was reported previously for its plant growth-promotion traits in sorghum and chickpea, and for its antagonistic potential against *Macrophomina phaseolina* that causes charcoal rot disease in sorghum in greenhouse and field screens^15,16^. Its PGP properties include the production of IAA, siderophore, lipase, cellulase, protease, chitinase, hydrocyanic acid (HCN), and β-1,3-glucanase. Besides these, it was also able to survive in moderate to extreme environmental conditions such as pH (5–11), salinity (0–6% NaCl), and temperature (20–40 °C)^15^.

*Amycolatopsis* was originally classified under the genus Nocardia^17^ but based on a combination of genotypic and phenotypic characters, this was reclassified under the genus *Amycolatopsis*^18^. It is a genus with high GC% and gram-positive bacteria belonging to the family Pseudonocardiaceae. These bacteria can produce different types of antibiotics and medicinally important metabolites due to this reason this genus is widely studied^19–21^. Due to the advanced and inexpensive genome sequencing techniques and growing clinical importance, 65 genome sequences of *Amycolatopsis* bacteria are currently publicly available in the NCBI genome database (https://www.ncbi.nlm.nih.gov/genome/). While *Amycolatopsis* genomes have been mined mainly for secondary metabolites^22^, not much information is available for novel genes/pathways underlying the PGP traits^23^. In the current investigation, the genome sequence of *Amycolatopsis* sp. BCA-696 was decoded to identify the BGCs and/or pathways associated not only with the PGP/biocontrol traits but also with some of the traits related to tolerance to pathogens. Besides, this strain was found to be distinct enough to be a separate species, and a pan-genome analysis of genomes of the *Amycolatopsis* genus was also conducted.

## Results

### *Amycolatopsis* sp. BCA-696 draft genome

In order to obtain the draft genome of this strain, sequencing was done using two types of libraries, i.e., paired-end (PE) and mate-pair (MP), yielding ~ 9.9 million PE reads of size 100 bases and ~ 6.2 million MP reads of size 250 bases, respectively, with approximate coverage of > 500× (assuming ~ 9.5 Mb as median assembly size of *Amycolatopsis* genomes available at NCBI).

**Figure 1:**
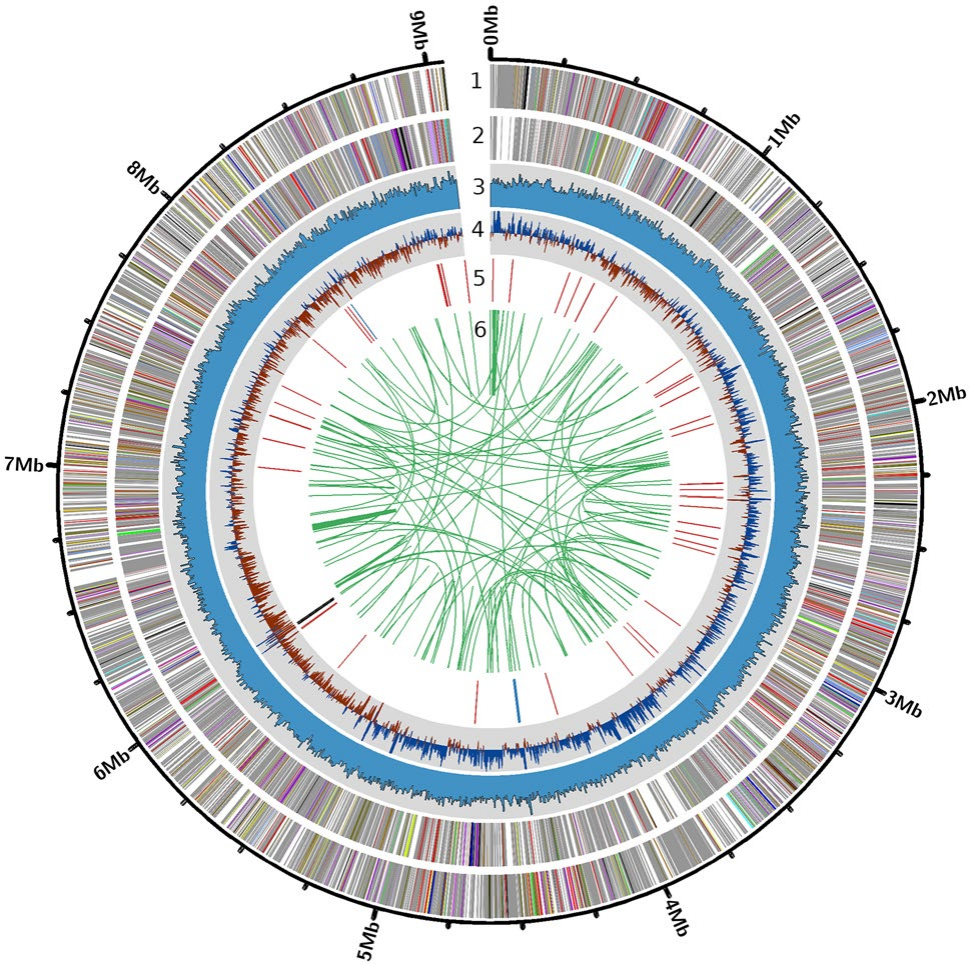
Visualization of different features on the *Amycolatopsis* sp. BCA-696 genome. Coding regions on forward and reverse strands are shown as 1st and 2nd concentric circular layers from outside(the colors differentiate the gene categories shown in Fig. 2), 3rd layer shows an average read depth in a 5 kb window along the genome, 4th layer shows GC skew (positive values in blue and negative values in brown color), 5th layer shows rRNAs (blue bands), tRNAs (red bands), and CRISPR spacers (black bands), and 6th layer shows similar repeats on the genome connected by lines (generated using Circos V.0.69.8).

The pre-processed reads were assembled de novo, initially generating 112 contigs, which were further ordered to obtain a single linear scaffolded genome sequence (Table 1, Supplementary Table S1). The genome sequence was of length 9,059,528 bp with GC content of 68.75% and a very nominal number of anonymous nucleotides (total size: ~ 6.5 Kb) (Fig. 1).

**Table 1:** General features of the *Amycolatopsis* sp. BCA-696 genome assembly.

Category	Information
Species	*Amycolatopsis* sp.
Strain	BCA-696
**Assembly details**	
Length (bp)	9,059,528
N50 value	9,059,528
Count of N's (percentage)	6567 (0.07%)
Count of N's per 100 kbp	72.49
GC percentage	68.75%
**Annotated genome features**	
Coding density percentage	90.34%
CDS count (mean length bp)	8716 (939.02)
tRNA count (mean length bp)	55 (75.93)
rRNA count (mean length bp)	6 (1465.5)
Repeat regions count (mean length bp)	213 (333.66)
CRISPR repeat (mean length bp)	42 (29)
CRISPR spacer count (mean length bp)	40 (33.62)
CRISPR array (mean length bp)	2 (1281.5)
**Protein features**	
Number of proteins with functional assignments (percentage)	5289 (60.66%)
Number of hypothetical proteins (percentage)	3427 (39.30%)
Number of proteins with Gene Ontology assignments	1329
Number of Proteins with subsystem assignments	1546
**Pangenome details**	
Number of core genes	3627
Number of unique genes	1423

To assess the completeness of the draft assembly, a reference set of 356 “Benchmarking Universal Single-Copy Orthologs” (BUSCOs) derived from 948 genomes from class actinobacteria lineage was created, and 355 (98.9%) BUSCOs were present in the *Amycolatopsis* sp. BCA-696 assembly. Out of the 355 BUSCOs identified, all of them were complete and just three of them were duplicated. Only one BUSCO was fragmented, indicating a high degree of completeness of the generated assembly.

### *Amycolatopsis* sp. BCA-696 genome annotation

**Figure 2:**
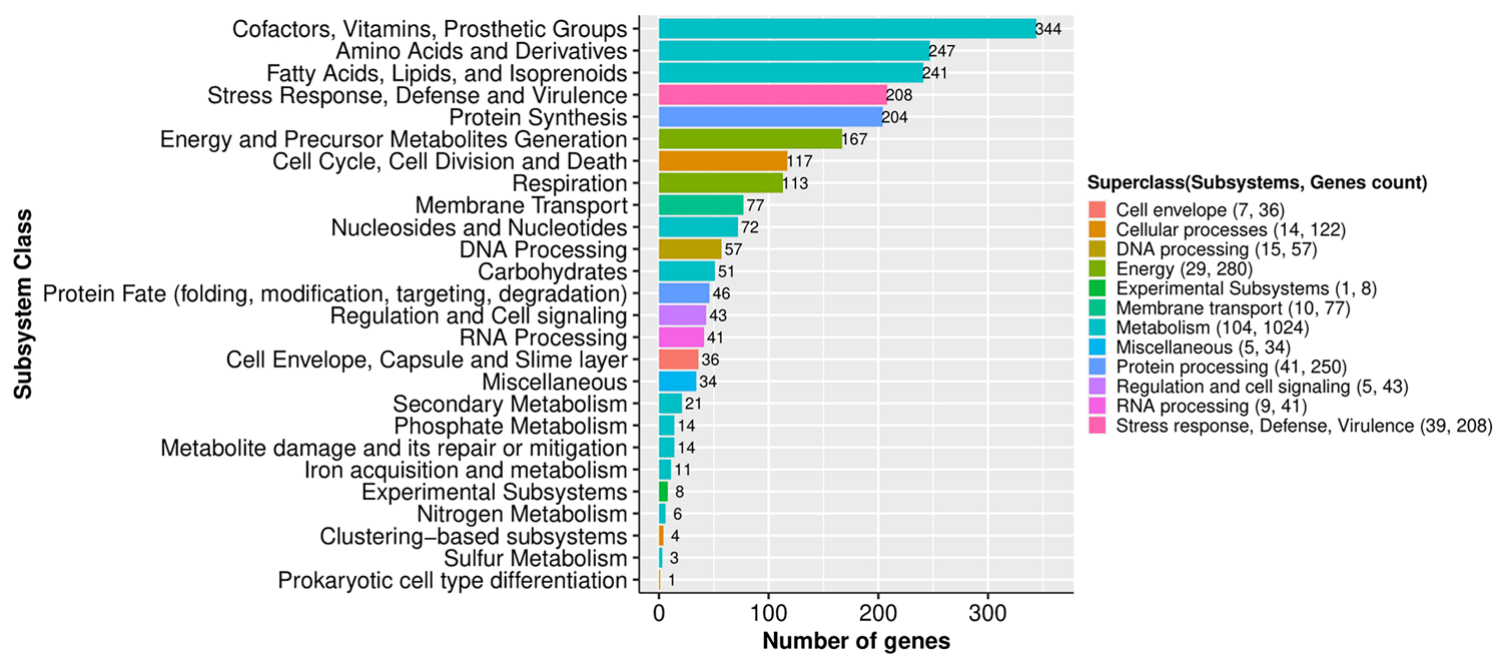
Distribution of subsystem classes in *Amycolatopsis* sp*.* BCA-696 genome (generated using ggplot2)***.***

The annotation of the genome assembly provided a total of 8,716 protein-encoding genes (PEGs), with an average length of 939.02 bp, occupying 90.34% of the genome (Table 1). The genome also contained 61 RNA coding regions, 213 repeat regions, 42 CRISPR repeats, 40 CRISPR spacer, and 2 CRISPR array regions. Out of 61 RNAs, the number of rRNA and tRNA genes were 6 and 55, respectively (Table 1). Among the predicted PEGs, 5289 (60.66%) proteins were assigned with functions and 3427 (39.30%) were hypothetical proteins. The cofactors, vitamins, prosthetic groups, and pigments (344 genes) found to be the most abundant subsystem class, followed by amino acids and derivatives (247 genes), fatty acids, lipids, and isoprenoids (241 genes), stress response, defense, and virulence (208 genes), protein synthesis (204 genes), energy and precursor metabolites generation (167 genes), cell cycle, cell division, and death (117 genes), respiration (113 genes), and membrane transport (77 genes), etc. (Fig. 2).

### Pan genome of *Amycolatopsis* species & genes specific to BCA-696

**Figure 3:**
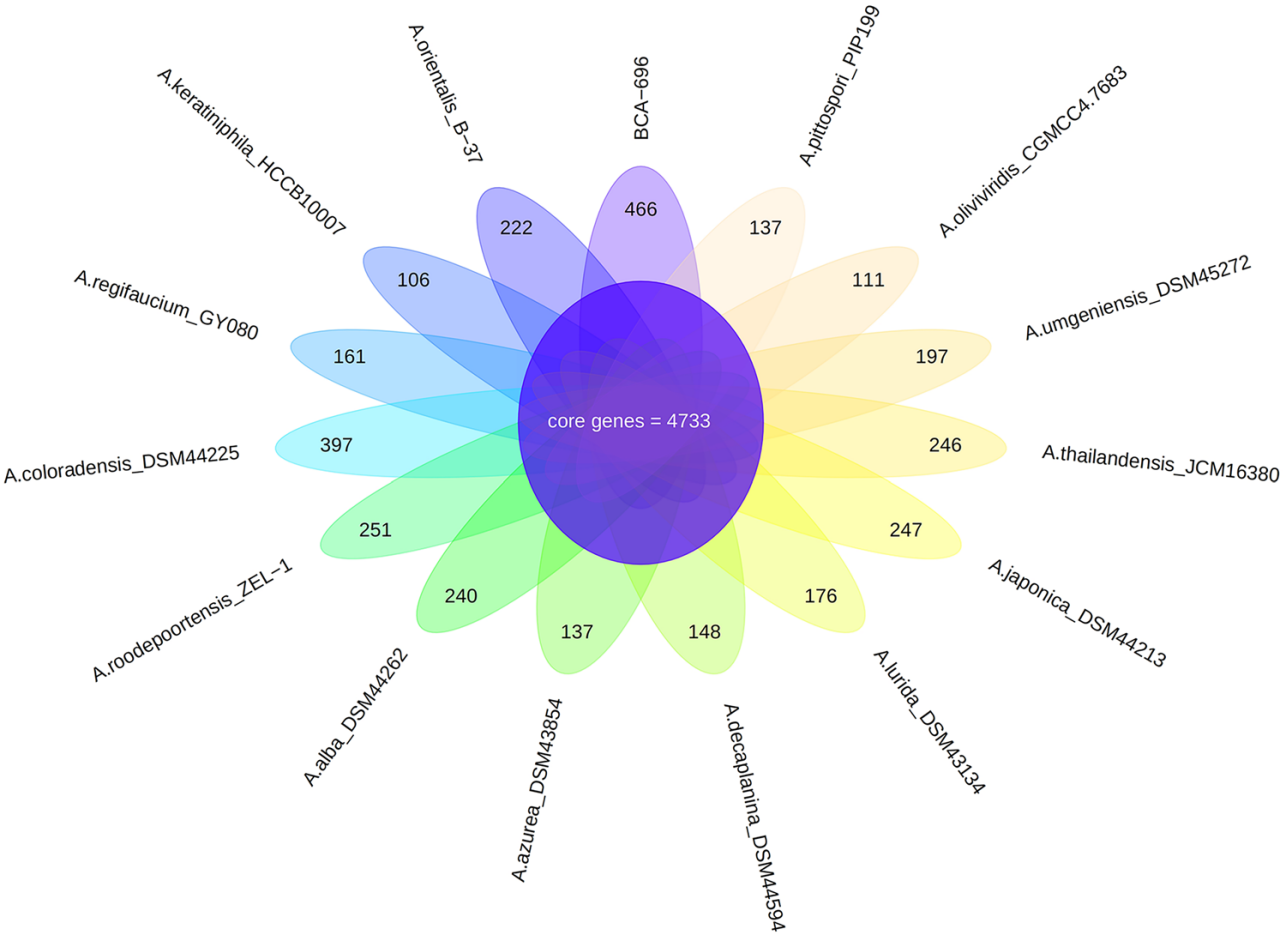
Flower plot showing Core genes and unique genes between fifteen closely related *Amycolatopsis* strains based on Orthofinder results. The number of core genes, shared by all of them, is shown in the center of the flower plot, and the unique genes for each strain are as flower petals (generated using rstudio using plotrix V.3.8-4) (for the list of type strains refer to “Materials and methods”).

In order to infer the genomic similarity or variation among the *Amycolatopsis* species, a pan-genome analysis was conducted. A pan-genome, comprising all genomes of the *Amycolatopsis* genus with a scaffold or higher level assembly (n = 76) demonstrated huge diversity in terms of gene composition: out of a whopping 375,299 orthologous groups, core genes were a much smaller set (< 1%) compared to the cloud genes (> 95%) (Supplementary Fig. S1). Since our focus was on genomic regions unique to *Amycolatopsis* sp. BCA-696, a subset comprising this genome and the genomes of fourteen closely related species/strains, all from a single clade within the pan-genome-based tree, were re-analyzed. A total of 35,318 orthologous gene clusters could be divided into a core genome of 3,627 (41.6%) orthologous gene clusters, having more than 99% similarity present across all fifteen strains, and the unique genes which ranged from 654 (in *A. keratiniphila)* to 2557 genes (in *Amycolatopsis coloradensis) *(Supplementary Fig. S2). The genome of *Amycolatopsis* sp. BCA-696 has 1423 (16.3%) strain-specific genes. Since as many as 1/6th (n = 1423) of total genes were reported as unique, this required a rigorous evaluation by using an algorithm that establishes orthology very accurately. Orthology search using Orthofinder pipeline^24^ reported a core set of 4,733 (42.6%) genes and 466 (4.2%) unique genes in *Amycolatopsis* sp. BCA-696 genome (Fig. 3). Out of these 466 unique genes, only 53 genes could be functionally annotated(by RAST pipeline^25^ and Reciprocal Best Blast) (Supplementary Table S2).

Among these 53 unique genes, one gene (Genbank ID: WYW18527.1) has been found to be involved in the biosynthesis of Bialaphos antibiotic (carboxyvinyl-carboxyphosphonate phosphorylase). Previously this antibiotic was reported only in Streptomyces species, possessing bactericidal, fungicidal, and herbicidal properties^26^. Other unique genes included drug antiporters, multiple transporter proteins, genes conferring resistance to antibiotic chloramphenicol and streptomycin, and endonucleases.

### Phylogenetic relationships of BCA-696

**Figure 4:**
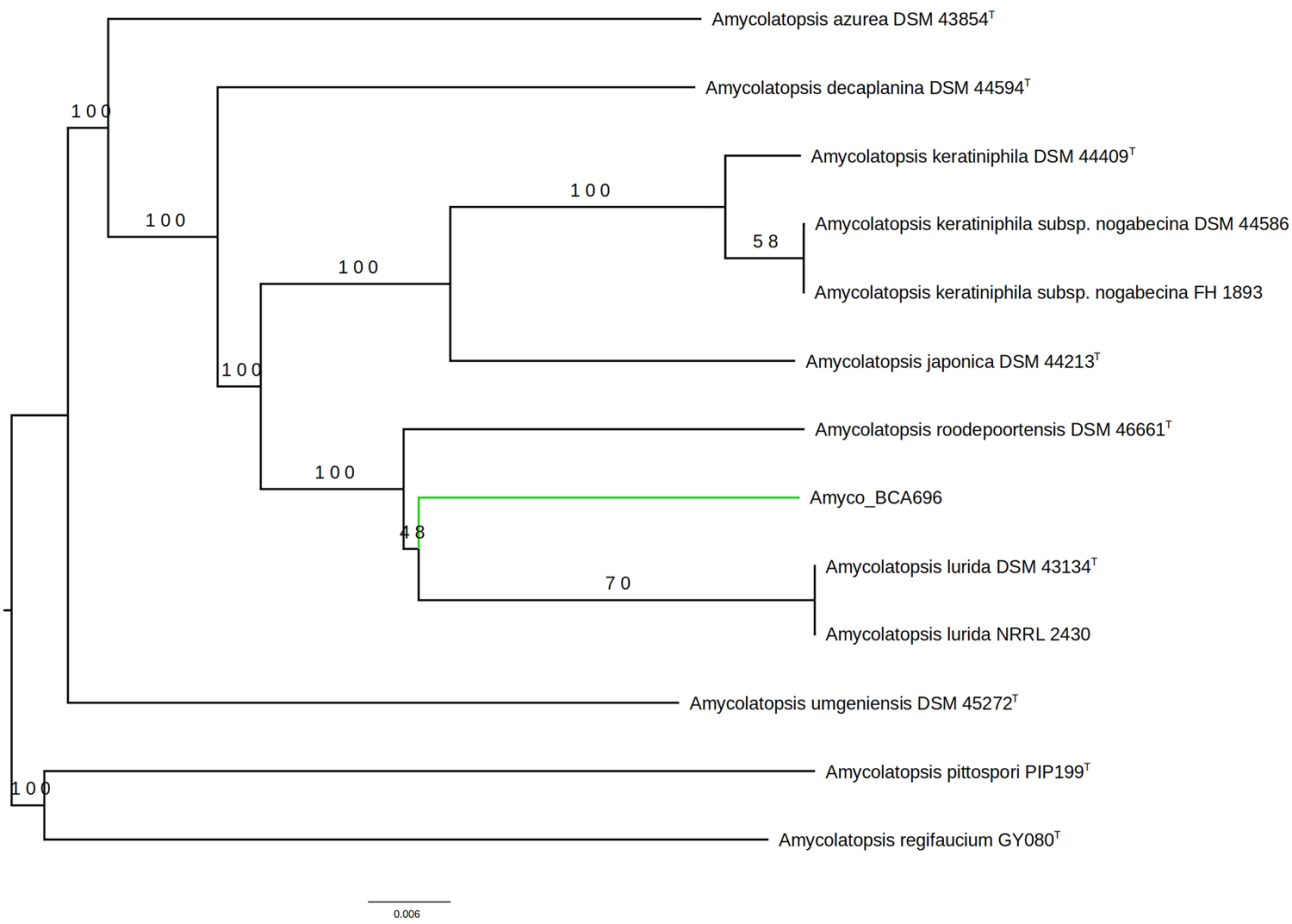
Phylogenetic tree obtained from TYGS server comparing genomes of Amycolatopsis BCA-696 with several closely related species/strains available in the online server (The type strains are indicated with a “T” in superscript). The TYGS analysis differed slightly from the results of Roary and Phylophlan3, showing A. roodepoortensis diverging from the common ancestors of A. lurida and Amycolatopsis sp. BCA-696. The bootstrap values (out of 100 iterations) are shown for each branch (generated from TYGS server https://tygs.dsmz.de/).

To establish the taxonomic positioning of BCA-696 within the *Amycolatopsis* genus, we used methods based on the overall genome relatedness index, which showed that *Amycolatopsis* sp. BCA-696 genome was closer to the genomes of *A. lurida* strains than any other *Amycolatopsis* genomes (Fig. 4). But the bootstrap value for the branch leading to BCA-696 was poor (~ 50 out of 100) showing the uncertainty in the position of BCA-696. Since the previous taxonomic assignment of this strain was based on 16S rRNA sequence, the availability of draft genome information of the strain has positioned it in the phylogenetic tree equidistant to *A. lurida* and *A. roodepoortensis* without being closer to either of them, suggesting this strain might be a new species.

### Biosynthetic gene clusters in *Amycolatopsis* sp*.* BCA-696 genome

**Figure 5:**
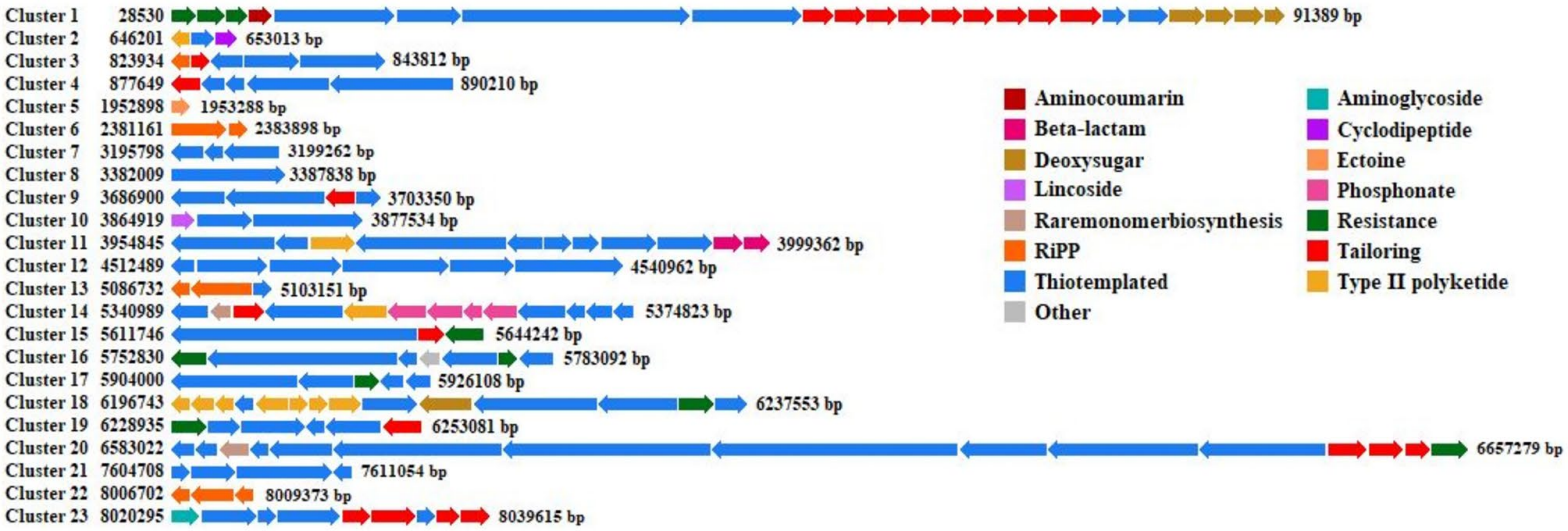
Physical maps of predicted biosynthetic gene clusters (BGCs) in the genome of *Amycolatopsis* sp. BCA-696. Arrows indicate the direction of gene transcription, while the chemical classes of products of the biosynthetic pathways encoded by the genes are indicated with different colors. The positions in the genome and the maps were generated from PRISM4 (https://prism.adapsyn.com/).

In its genome of size ~ 9 Mb, 23 to 35 Biosynthetic Gene Clusters (BGCs) were predicted by two widely used BGC predictors (Fig. 5, Supplementary Table S3, Supplementary Fig. S3). The commonly observed BGC classes were ‘Resistance’, ‘Tailoring’, ‘Thiotemplated’, ‘Type II polyketide’, ‘ribosomally synthesized and post-translationally modified peptide product’ (RiPP), ‘phosphonate’, etc. Whether any of the genes unique to BCA-696 overlap with these BGCs, a comparison of their genomic coordinates did not show any overlap.

A detailed examination of core and additional biosynthetic genes showed two large clusters for the biosynthesis of two antibiotics- vancomycin and enediyne, spread within genomic coordinates 36,435—96,579 and 3,372,806—3,960,062, respectively (Supplementary Table S3). A full pathway for vancomycin biosynthesis was observed, however, for the Enediyne family of antibiotics, pathways were observed for the biosynthesis of its core molecule (neocarzinostatin) necessary for further reactions, and one of its derivatives, maduropeptin.

### Genes underlying PGP and bio-control traits/pathways

The *Amycolatopsis* sp*.* BCA-696 genome assembly was analyzed to identify the genes involved in PGP and biocontrol activities. The strain under this study was experimentally validated to produce metabolites such as siderophores and hydrocyanic acid and shows enzymatic activity for cellulase, chitinase, lipase, indole acetic acid, and 1,3-beta-glucanase ^16^.

Among the siderophores, evidence for the presence of complete biosynthetic pathways for the production of catecholate or mixed types siderophores namely, bacillibactin, enterochelin, mycobactin, etc., were examined in the genome annotation of *Amycolatopsis* sp. BCA-696. In the RAST annotation, while the enzymes for biosynthesis of siderophore precursors and transporters involved in the export/import of siderophore or Fe-siderophore complex were found (Supplementary Table S4), the genes for biosynthesis of these siderophores from their precursors were largely missing in the (RAST) annotation. Orthology-based search only showed the presence of partial pathways (Supplementary Table S5, Supplementary Fig. S4).

Coming to the biocontrol traits, presence of three key cellulolytic enzymes was observed in *Amycolatopsis* sp. BCA-696 assembly: Endoglucanase (EC 3.2.1.4), Beta-glucosidase (EC 3.2.1.21), and Cellulose-1,4-beta-cellobiosidase (EC 3.2.1.91; Exoglucanase), wherein the former-two were present in many copies, ranging up to nine (Supplementary Fig. S5, Supplementary Table S6). Regarding the Chitinase activity, all enzymes related to the Chitin degradation pathway were also identified in *Amycolatopsis* sp. BCA-696 assembly which are: chitinase (EC 3.2.1.14), beta-*N*-acetylglucosaminidase, chitin deacetylase, chitosanase, exo-1,4-beta-d-glucosaminidase (Supplementary Fig. S6, Supplementary Table S7). Like other hydrolytic enzymes, bacterial lipases also play an important role in biocontrol against many phytopathogens, and also in inducing plant defence response ^27^. Two enzymes namely, lipase (EC 3.1.1.3) and Diacylglycerol *O*-acyltransferase (EC 2.3.1.20), were identified in the *Amycolatopsis* sp*.* BCA-696 assembly, indicating the presence/absence of underlying genes (Supplementary Fig. S7, Supplementary Table S8).

Examination of biosynthetic genes/enzymes for auxin (IAA) showed the presence of the pathway involving the Tryptamine intermediate. For alternate pathways (for auxin biosynthesis), although a few genes were also present, ultimately the pathways were incomplete (Supplementary Table S9, Supplementary Fig. S8).

## Discussion

### First genome assembly of an agriculturally important species from the *Amycolatopsis* genus:

*Amycolatopsis* genus is well documented for its antibiotic-producing traits^28,29^ and thus is an obvious focal point for drug discovery programs. To understand the genomic basis behind this(i.e., secondary metabolite production), 150 *Amycolatopsis* genomes have so far been sequenced (based on data accessed in mid-2023) and about one-fifth of them are either complete or at the chromosome level assembly. The importance of *Amycolatopsis* in the agricultural sector is, however, not well known. Perhaps, we were the first to report its usefulness as PGP in sorghum and chickpea^15^, and its antagonistic potential against *M. phaseolina* mediated charcoal rot disease in sorghum^16^. Along a similar line, to uncover the genomic components underlying the PGP/biocontrol traits, we reported chromosome-level genome assembly of BCA-696 strain: ~ 9.06 Mb in size with 8,716 protein-coding genes (Fig. 1, Table 1).

### Phylogenomic analysis indicated that *Amycolatopsis* sp. BCA-696 is a species on its own

The taxonomic classification of the BCA-696 strain has been available only till the genus level. The analysis using the whole genome sequence showed that this strain is closer to *A. lurida* (Fig. 4). However, the poor bootstrap value for the branch of BCA-696 implied that the species-level classification of BCA-696 is still elusive with the existing genomic information.

### Like several *Amycolatopsis* genomes, the *Amycolatopsis* sp. BCA-696 too had many unique genes

The *Amycolatopsis* genomes, in particular the BGCs, have been compared in multiple studies^22,29,30^. A comparison involving 41 genomes showed a core set of size 1212 genes^29^, which was almost one-third of the size observed in this study (n = 4733), and the difference can be attributed mainly to the different number of genomes involved (41 versus 15). Given the diversity in the biosynthetic potential of secondary metabolites among *Amycolatopsis* species, the accessory and unique (gene) sets attain a higher importance than the core set. While comparing the Gene Cluster Families (GCFs) in 41 genomes, the conserved features accounted for only a small proportion, and a vast number of GCFs (67%) were represented by a single genome^29^. In the BCA-696 strain, a prediction of unique features gave 466 genes (Fig. 3), which corroborates earlier reports of a significant fraction of unique genes present in *Amycolatopsis* genomes.

### Unique genes of *Amycolatopsis* sp. BCA-696 were enriched with antibiotic biosynthesis and resistance functions

While the functional annotation of the majority of the unique genes was unavailable, among the annotated genes antibiotic-related functions were the most prominent. It contained the gene for the biosynthesis of Bialaphos antibiotic (Supplementary Table S2), which has not been reported yet among *Amycolatopsis* species, and just one *Streptomyces* species has been known to have capability^31,32^. This antibiotic has been reported to have fungicidal and herbicidal roles, thus a potential biocontrol agent. In addition, several antibiotic transporters were also found which may play a role in secretion or defense (Supplementary Table S2). Another peculiar feature was the presence of Chloramphenicol and Streptomycin phosphotransferase which are involved in providing resistance against those antibiotics (Supplementary Table S2).

### The genes identified for PGP traits indicated inhibition of *M. phaseolina* potentially due to the hydrolytic enzymes and antibiotics

Plant-growth-promoting bacteria isolated from the rhizosphere are known to produce growth hormones such as auxins and siderophores and hydrolytic enzymes such as chitinase, cellulase, and β-1,3-glucanase and help plants to inhibit pathogens either directly or indirectly^33–35^. *Amycolatopsis* BCA-696 has been reported to produce biocontrol and PGP traits including siderophore, HCN, chitinase, protease, cellulase, β-1,3-glucanase, lipase, and IAA under in vitro conditions^15^. The genome sequence analysis indicated that this strain can potentially biosynthesize auxin only through the Tryptamine pathway (Supplementary Table S9) and may need to cooperate with other rhizosphere bacteria for alternate biosynthesis pathways. Moreover, for Cellulase, Lipase, and Chitinase, complete biosynthesis pathways were identified (Supplementary Table S6–S8). Besides, complete or partial biosynthesis pathways for the diverse siderophore molecules were identified along with a number of transporters (Supplementary Tables S3–S5). The presence of biosynthetic genes for the Enediyne family of antibiotics (Supplementary Table S3), which typically act by DNA cleavage^36^, has been an interesting finding in BCA-696, as some of these secondary metabolites have earlier been shown for antifungal activity among diverse plant and animal pathogens ^37,38^. Hence, it is concluded that *Amycolatopsis* BCA-696 potentially produces hydrolytic enzymes or antibiotics that have the potential to inhibit the pathogen *M. phaseolina* that causes charcoal rot in sorghum.

### Future Directions

The﻿ usefulness of *Amycolatopsis* BCA-696, for biocontrol of charcoal rot disease in sorghum, has been demonstrated at both greenhouse and field conditions in our previous study^16^. We also reported the tolerance of BCA-696 on a wide range of pH (5–11), temperatures (20–40 °C), NaCl concentrations (0–6%), and fungicides (including Bavistin up to 2500 ppm, Thiram up to 3000 ppm, Benlate up to 4000 ppm, Captan up to 3000 ppm and Ridomil up to 3000 ppm^15^. These traits could help BCA-696 to survive in harsh environments under natural conditions and thus this bioagent can be used in integrated disease management programs. Further, *Amycolatopsis* BCA-696 needs to be formulated as a bio-inoculant and used for the biocontrol of charcoal rot in other crops. The secondary metabolite(s) responsible for the inhibition of *M. phaseolina* need to be experimentally characterized. In the absence of a high level of genetic resistance in high-yielding varieties, *Amycolatopsis* BCA-696 could be effective in controlling charcoal rot disease and related loss in grain and stover quality of sorghum.

## Material and methods

### Source of actinobacterial strain

A strain of *Amycolatopsis* sp. BCA-696 (GenBank accession number of 16S ribosomal RNA gene: KM191337), previously reported by us to have the capacity for PGP in sorghum and chickpea^15^ and for its antagonistic potential against *Macrophomina phaseolina* that causes charcoal rot disease in sorghum^16^ was selected for the present study.

### Isolation of DNA

DNA of *Amycolatopsis* sp. BCA-696 was isolated as per the protocols mentioned in Gopalakrishnan et al., 2020^14^. In brief, BCA-696 was inoculated in starch casein broth and incubated for 120 h at 28° C. At the end of incubation, the culture was centrifuged at 10,000*g* for 10 min at 4 °C and the cells were washed twice with STE buffer (sucrose 0.3 M, Tris/ HCl 25 mM and Na_2_EDTA 25 mM, pH 8.0). The supernatant was discarded but the pellet (1 g) was re-suspended in 8.55 ml STE buffer and 950 µl lysozyme (20 mg/ml STE buffer) and incubated for 30 min at 30 °C. This was followed by the addition of 500 µl of SDS (10%; w/v) and 50 µl of protease (20 mg/ml) and the mixture was held at 37 °C for one h. At the end of incubation, 1.8 ml of NaCl (5 M) was added with gentle mixing to avoid shearing the DNA, and 1.5 ml of CTAB (10%; w/v) in 0.7 M NaCl (CTAB/NaCl solution) and incubated for 20 min at 65 °C. Once CTAB was added, all the remaining steps were carried out at room temperature. The lysate was extracted twice with an equal volume of phenol/chloroform/isoamyl alcohol (25:24:1; by vol) and centrifuged at 13,000*g* for 10 min. Finally, the aqueous phase was extracted with chloroform/isoamyl alcohol (24:1, by vol) and transferred to a tube followed by the addition of 600 µl of propan-2-ol and DNA spooled out after 10 min. Alternatively, it was recovered by centrifugation at 12,000*g* for 10 min. The pellet was washed twice with ethanol (70%; v/v), vacuum dried, and dissolved in 2 ml of TE buffer (10 mM Tris/HCl and 1 mM EDTA, pH 8.0). RNase A (50 mg/ml) was added with incubation at 37 °C for 2 h. The sample was again extracted with phenol as described above. DNA was re-precipitated from the aqueous phase with the addition of 100 µl of 3 M sodium acetate (pH 5.3) and 600 µl of propan-2-ol. The DNA pellet was washed with ethanol (70%; v/v), dried, and dissolved in TE buffer. The purity of the BCA-696 DNA was checked in the agarose gel electrophoresis and quantified using NanoDrop.

### Whole genome sequencing

About ~ 5 μg high-quality genomic DNA (free from any contaminant and having A260/280 ratio in the range of ~ 1.8 to 2.0 with DNA concentration ≥ 100 ng/μl) was sent to AgriGenome Labs (Kochi, India) for library preparation and next-generation sequencing using the Illumina platform. The Genomic DNA was fragmented, and a paired-end library with insert size 300 bp, and a mate-pair library with insert size 5 Kbp, were prepared. The DNA fragment libraries were validated by tapestation, and sequenced ~ 9.905 million paired-end (100 bp × 2) and ~ 6.242 million mate pair (250 bp × 2) reads on the Illumina HiSeq 2500.

### Genome assembly and its assessment

The Whole-genome sequenced paired and mate-pair reads of the bacterial genome were cleaned (by removing adapters, primer sequences, etc.) using Trimmomatic V.0.39^39^. The cleaned reads were de novo assembled using SOAPdenovo V2.04 and SPADES V3.10.1 assemblers^40,41^. Two assemblies were assessed and compared by using QUAST V5.0.2 ^42^ followed by discarding the contigs having < 500 bp length and coverage < 5. The GapCloser V1.0.1 ^43^ was used to close the gaps that emerged during the scaffolding process by the de novo assembler, using the abundant pair relationships of short reads. Further contigs were subjected to Pathosystems Resource Integration Center (PATRIC V3.6.9)^44^ and KmerFinder V3.2 ^45^ search to find the closely related genomes. In order to do reference genome-based reordering of contigs *Amycolatopsis albispora, Amycolatopsis keratiniphila, Amycolatopsis japonica, and Amycolatopsis orientalis* genome sequences were obtained from NCBI microbial genomes database followed by scaffolding using Multidraft-Based Scaffolder (MEDUSA V1.6)^46^. The resulting scaffolds were subjected to an NCBI BLAST database search to check the contamination and scaffold hits other than the *Amycolatopsis* genus were discarded. The completeness of the genome assembly was examined using the tool ‘Benchmarking Universal Single-Copy Orthologs (BUSCOs) V5.1.3 ^47^. The Circos plot showing the features on the genome (Fig. 1) was generated using Circos V.0.69.8 ^48^.

### Genome annotation

The annotation of the assembled genome was carried out by Rapid Annotation using the Subsystem Technology (RAST) server (http://rast.nmpdr.org/rast.cgi), through the RASTtk pipeline^25^ (Accessed on: April 2020). The genome-based phylogenetic relationships between the BCA-696 strain and its closely related species used to reorganize scaffolds were determined using the reference sequence ALignment-based PHYlogeny (REALPHY) builder method^49^ (accessed on: April 2020). Figure 2 showing the subsystem classes in BCA-696 was plotted using ggplot2 package^50^ from Rstudio.

### Comparative analysis of *Amycolatopsis* sp. BCA-696 genome

The pangenome analysis was performed using all *Amycolatopsis* genomes at the NCBI database with at least scaffold level completion (n = 76, Supplementary Table S10) using Roary pipeline v3.13.0^51^. Having observed an unusually small core set size (< 1%) and a whoppingly large cloud gene set size (> 95%), the pan-genome analysis was repeated on a subset of genomes that were closely related to BCA-696. Species/strains belonging to the clade, identified from the pan-genome-based tree, in which BCA-696 was also present were selected as close relatives. This set (of closely related species/strains) was augmented by including a few more species/strains having contig-level completion. The fourteen close relatives of BCA-696 were: *A. orientalis strain B-37* (GCF_000943515.2)*, A. keratiniphila strain HCCB10007* (GCF_000400635.2)*, A. regifaucium strain GY080*^*T*^ (GCF_001558125.2)*, A. coloradensis strain DSM 44225*^*T*^ (GCF_001953865.1)*, A. roodepoortensis strain ZEL-1* (GCF_024628825.1)*, A. alba strain DSM 44262*^*T*^ (GCF_000384215.1)*, A. azurea strain DSM 43854*^*T*^ (GCF_001995215.1)*, A. decaplanina strain DSM 44594*^*T*^ (GCF_000342005.1)*, A. lurida strain DSM 43134*^*T*^ (GCF_000749465.2)*, A. japonica strain DSM 44213*^*T*^ (GCF_000732925.1)*, A. thailandensis strain JCM 16380*^*T*^ (GCF_002234405.1)*, A. umgeniensis strain DSM 45272*^*T*^ (GCF_014205155.1)*, A. oliviviridis strain CGMCC 4.7683* (GCF_014654365.1)*,* and *A. pittospori strain PIP199*^*T*^ (GCF_013870525.1) (The type strains are indicated with a “T” in superscript). The genome sequences were annotated with the rapid prokaryotic genome annotation tool Prokka V1.14.6^52^ and implemented Roary pipeline on input annotation files (in GFF format) with a minimum 95% percentage identity for BLASTp. Core gene sequences were aligned with Multiple Alignments using Fast Fourier Transform (MAFFT V7.520)^53^, and the CD-HIT V4.7^54^ algorithm used to perform clustering was embedded in the Roary pipeline. Further analysis was performed on the gene presence/absence file generated by the Roary analysis.

### Orthology-based re-analysis of BCA-696 specific genes

Orthofinder (version 2.5.4)^24^ was used with default parameters to find common ortholog groups among BCA-696 and 14 other genomes of *Amycolatopsis* species taken from the NCBI database (Acessed on: October 2022). Genes unique to BCA-696 were selected and mined for functional details using the KEGG PATHWAY Database (Accessed on: October 2022)^55^ (https://www.genome.jp/kegg/pathway.html).

### Phylogenetic relationships of BCA-696

To obtain taxonomic positioning of *Amycolatopsis* BCA-696 strain in the *Amycolatopsis* genus based on the genome sequences, the BCA-696 genome was submitted to the Type (Strain) Genome Server (TYGS) (accessed on: Jan 2024)^31^, and the ten closest genomes were selected by the TYGS to construct the phylogenetic tree with default setting. The TYGS uses overall genome relatedness index (OGRI) methods (GBDP & dDDH).

### Prediction of biosynthetic pathways (BGCs) for secondary metabolites and PGP traits

PRISM4 (accessed on: July 2020)^56^ was used to mine BGCs in the BCA-696 genome. It allows the prediction of a BGC's location and provides insights into the putative structure of the encoded secondary metabolite. BGCs were additionally predicted using another tool namely antiSMASH v7.0.0 ^57^ using 'relaxed' strictness level. Since none of the two tools gave precise details of the secondary metabolites actually being synthesized by the core and/or additional biosynthetic genes, protein sequences of these genes were searched in the KEGG ortholog/pathway database using ‘Automatic KO assignment and KEGG mapping service’ (BlastKOALA). Representative pathways for biosynthesis of Vancomycin and Enediyne from *Amycolatopsis keratiniphila*, for instance, were accessed at URLs https://www.genome.jp/pathway/aoi01055 and https://www.kegg.jp/pathway/aoi01059, respectively.

Further, pathway information of the PGP traits was obtained from the KEGG PATHWAY Database (Accessed on: July 2020)^54^, which provided details of the enzymes involved**.** In order to find orthologs of these KEGG enzymes in the genome of *Amycolatopsis* sp. BCA-696, the Reciprocal Best BLAST (RBB) search was performed between the proteomes of *Amycolatopsis* sp. B-696 and a few reference bacterial species (such as *E. coli*), already have orthologs (KO) of KEGG enzymes.

### Supplementary Information

Supplementary Information.

## Data Availability

The sequencing data generated in this study has been submitted to the National Centre for Biotechnology Information (NCBI) under the Bioproject ID: PRJNA765508. The genome assembly and annotation can be accessed from NCBI (Genbank accession number CP150484). The genome annotation by the RAST server can be accessed from the author's website (http://sls.uohyd.ac.in/new/fac_details.php?fac_id=34#DataandSoftwares).
