# Supplementary material for: Genome assembly, comparative genomics, and identification of genes/pathways underlying plant growth-promoting traits of an actinobacterial strain, Amycolatopsis sp. (BCA-696)
Source: Sci Rep. 2024 Jul 10;14:15934. doi: 10.1038/s41598-024-66835-y (PMC11237095; doi:10.1038/s41598-024-66835-y)
Supplement: Supplementary file 1 — Supplementary Information. [file 41598_2024_66835_MOESM1_ESM.pdf]

Title:

Genome assembly, comparative genomics, and identification of genes/pathways underlying plant growth-promoting traits of an actinobacterial strain, Amycolatopsis sp. (BCA-696)

Prasad Gandham[1,5^], Nandini Vadla[2^], Angeo Saji[2^], Vadlamudi Srinivas[1], Pradeep Ruperao[1], Sivasubramani Selvanayagam[1], Rachit K Saxena[1,3], Abhishek Rathore[1,4*], Subramaniam Gopalakrishnan[1,6*] and Vivek Thakur[2,*]

[1]International Crops Research Institute for the Semi-Arid Tropics (ICRISAT), Hyderabad, India
[2]Dept. of Systems & Computational Biology, School of Life Sciences, University of Hyderabad, Hyderabad, India
[3]Gujarat Biotechnology University, Gandhinagar, Gujrat, India
[4]Excellence in Breeding, International Maize and Wheat Improvement Center (CIMMYT), Hyderabad, India
[5]School of Plant, Environmental and Soil Sciences, Louisiana State University Agricultural Center, LA, United States. Mobile: +1 225 371 1937
[6]International Institute of Tropical Agriculture (IITA), Dar es Salaam, Tanzania

^= Joint first authors; Contributed equally.
*= Corresponding authors: abhishek.rathore@cgiar.org; vivek22@uohyd.ac.in; s.gopalakrishnan@cgiar.org

Supplementary figures

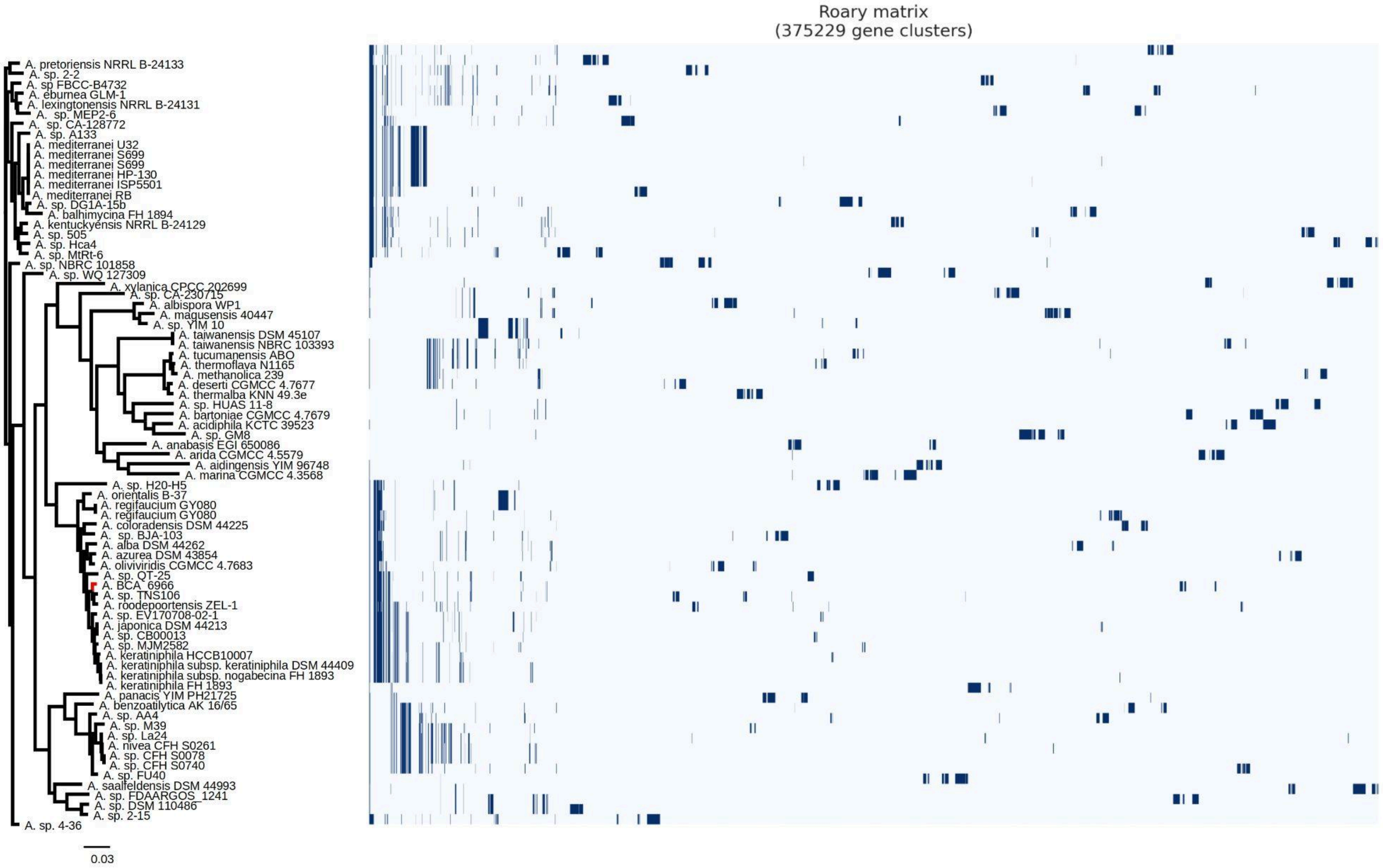

***Figure S1 (above)**: Pangenome matrix of 77 Amycolatopsis genomes with scaffolds or higher level assembly (list in Supplementary Table S10). The branch leading to Amycolatopsis BCA-696 has been highlighted in red, and most of the species from that clade were part of the subset of 14 which were intensively compared. The heatmap depicts the presence or absence of genes across the 77 species (generated using python script Roary_plots.py (https://github.com/sanger-pathogens/Roary/blob/master/contrib/roary_plots/roary_plots.py)).*

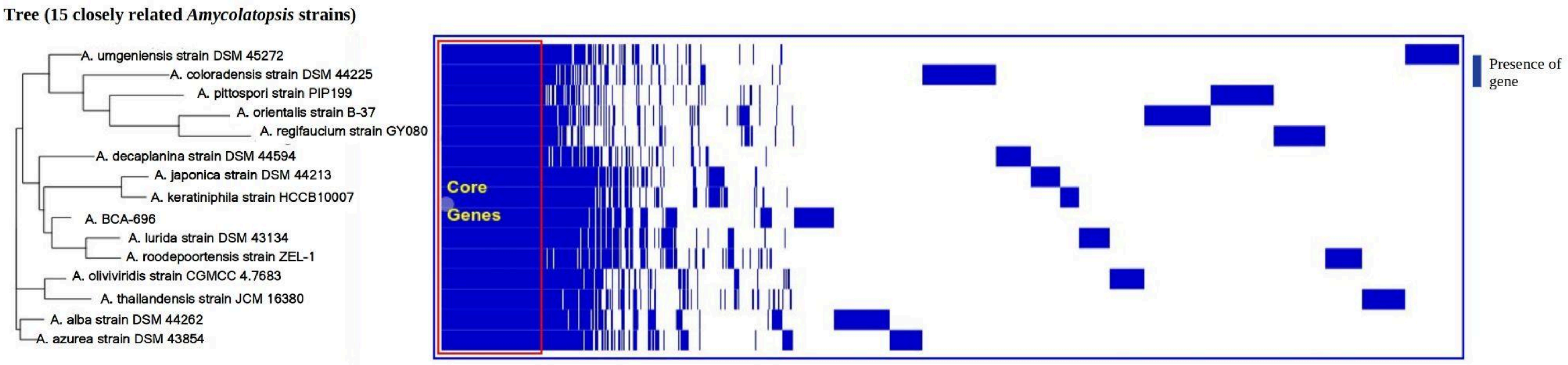

***Figure S2:** Phylogenetic relationship among related strains of Amycolatopsis. 15 closely related strains and their phylogenetic relationships are shown in the Cladogram(constructed using the Maximum Likelihood approach with the core genes) and the heatmap depicts the presence or absence of genes across the pangenome of 15 species (generated using rstudio package "pheatmap" V.1.0.12).*

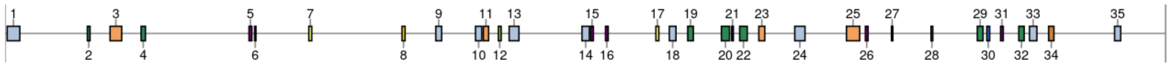

Region	Type	From	To	Most similar known cluster
Region 1	NRPS, NRPS-like, T3PKS	16,436	114,709	
Region 2	CDPS	642,327	663,013	
Region 3	T1PKS, hglE-KS	819,616	910,210	
Region 4	NAPAA	1,061,693	1,095,595	
Region 5	terpene	1,904,080	1,926,239	
Region 6	ectoine	1,947,899	1,958,288	
Region 7	lanthipeptide-class-iii	2,371,162	2,393,714	
Region 8	thiopeptide	3,098,548	3,122,183	
Region 9	T1PKS, RiPP-like	3,362,010	3,407,838	
Region 10	LAP, PKS-like, T1PKS, thiopeptide	3,672,224	3,721,006	
Region 11	PKS-like	3,733,536	3,774,540	
Region 12	amglyccycl	3,850,828	3,872,102	
Region 13	T1PKS, NRPS-like, NAPAA	3,934,846	4,011,235	
Region 14	NRPS, T1PKS	4,503,386	4,560,962	
Region 15	terpene	4,571,224	4,592,348	
Region 16	terpene	4,686,875	4,707,837	
Region 17	lanthipeptide-class-ii	5,080,327	5,103,152	
Region 18	terpene, NAPAA	5,185,325	5,237,103	
Region 19	NRPS	5,328,918	5,372,658	
Region 20	NRP-metallophore, NRPS	5,591,747	5,655,378	
Region 21	hydrogen-cyanide	5,671,999	5,684,825	
Region 22	NRP-metallophore, NRPS	5,734,234	5,793,470	
Region 23	T1PKS	5,884,001	5,930,423	
Region 24	T2PKS, T1PKS, NRPS	6,164,483	6,244,319	
Region 25	T1PKS	6,567,920	6,671,343	
Region 26	terpene	6,715,442	6,736,515	
Region 27	RiPP-like	6,919,203	6,930,027	
Region 28	aminopolycarboxylic-acid	7,228,244	7,241,613	
Region 29	NRPS	7,589,335	7,634,092	
Region 30	redox-cofactor	7,663,876	7,685,913	
Region 31	terpene	7,771,080	7,792,009	
Region 32	NRPS-like	7,913,026	7,955,476	
Region 33	lassopeptide, T1PKS	7,996,703	8,054,602	
Region 34	arylpolyene	8,146,283	8,187,434	
Region 35	other, PKS-like, amglyccycl	8,660,779	8,708,930	

Figure S3: *The Biosynthetic Gene Clusters (BGCs) predicted by antiSMASH using 'relaxed' setting. The panel on the top shows location of 35 BGCs in the genome. Below that shows the location of each BGC along with its annotation (i.e., functional classes). Expansion of abbreviations:- **NRPS**: Non-Ribosomal Peptide Synthesis, **T[123]PKS**: Type I/II/III PKS (Polyketide synthase), **CDPS**: tRNA-dependent cyclodipeptide synthases, **hglE-KS**: Heterocyst glycolipid synthase-like PKS, **NAPAA**: Non-alpha poly-amino acids like e-Polylysin, **RiPP**: Ribosomally synthesised and post-translationally modified peptide product (RiPP), and **LAP**: Linear azol(in)e-containing peptides (generated using Antismash V.7.0.0)*

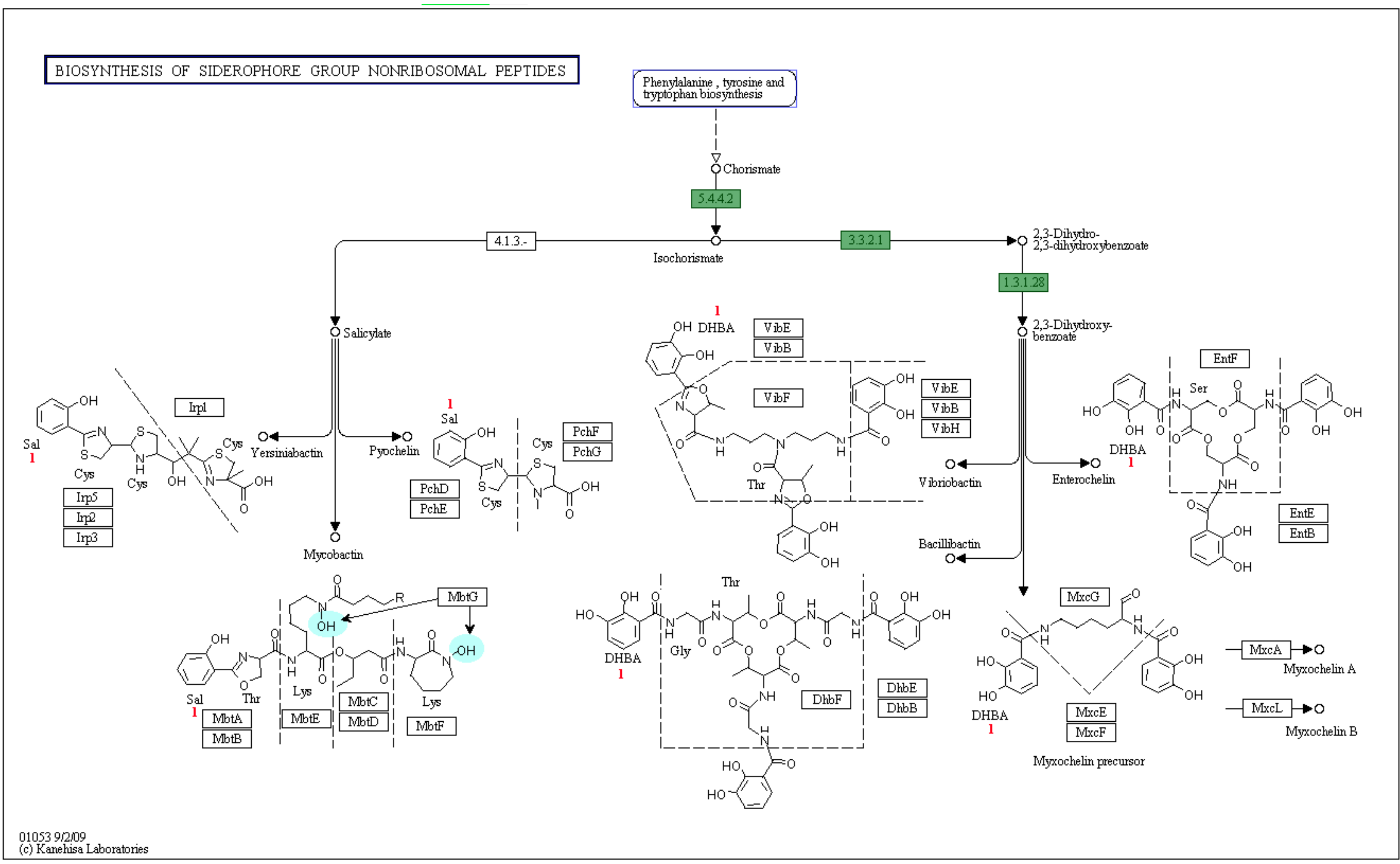

Figure S4: *Pathway of biosynthesis of different types of Siderophores in Amycolatopsis* sp. BCA-696. *The green-colored boxes in the siderophore pathway indicate the presence of genes in the assembly (based on RAST annotation) (generated from KEGG Database* https://www.kegg.jp*).*

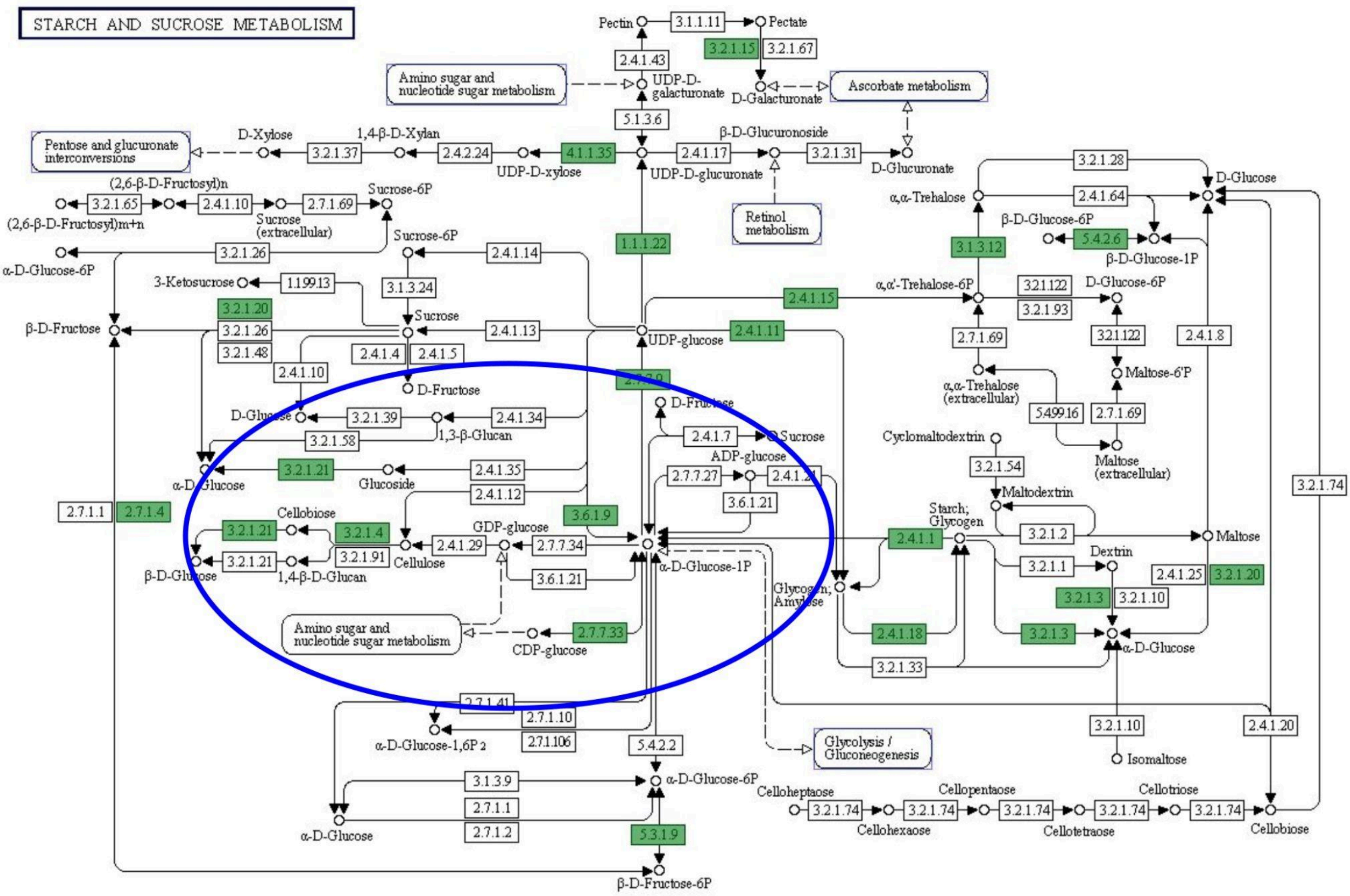

Figure S5: *Cellulose metabolism pathway (highlighted in blue circle). The green boxes in indicate the genes (or enzymes) present in Amycolatopsis* sp. BCA-696 (based on RAST annotation) *(generated from KEGG Database* https://www.kegg.jp*).*

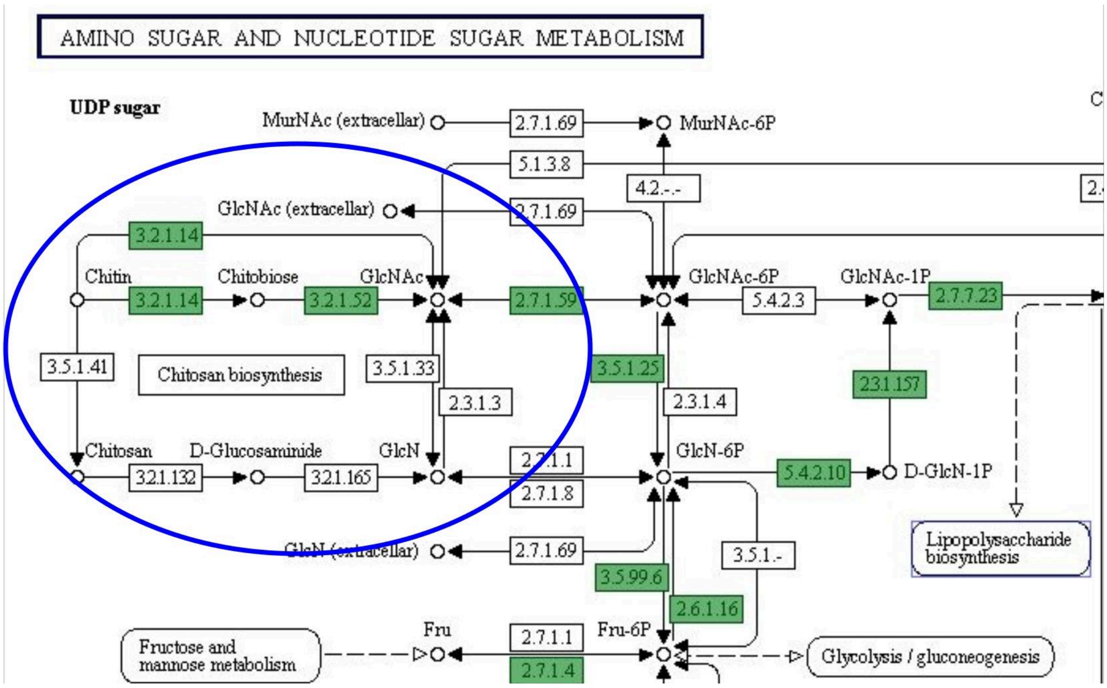

Figure S6: *Chitin degradation pathway (highlighted in blue circle). The boxes highlighted in green color indicate the enzymes present in the Amycolatopsis* sp. BCA-696 *assembly (based on RAST annotation) (generated from KEGG Database* https://www.kegg.jp*).*

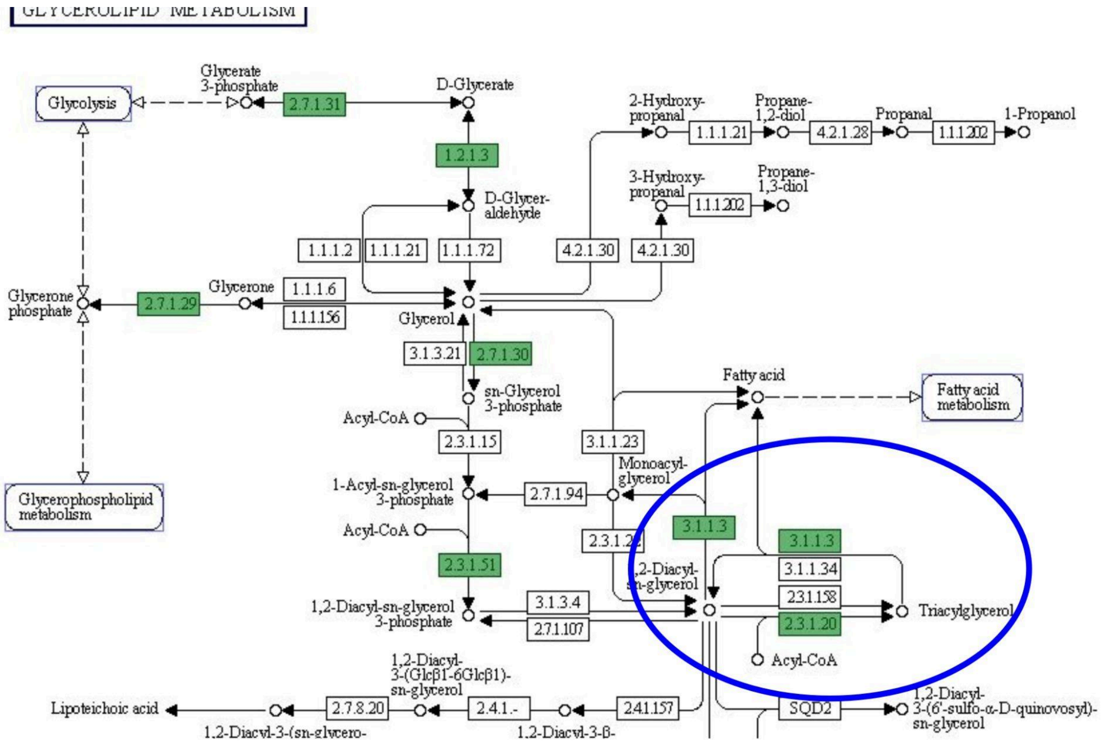

Figure S7: *Glycerolipid metabolism enzymes in Amycolatopsis* sp. BCA-696 *(highlighted in green color) indicates presence of lipases (highlighted with blue circle). The data is based on RAST annotation, and the pathway map was generated from KEGG Database (*https://www.kegg.jp*).*

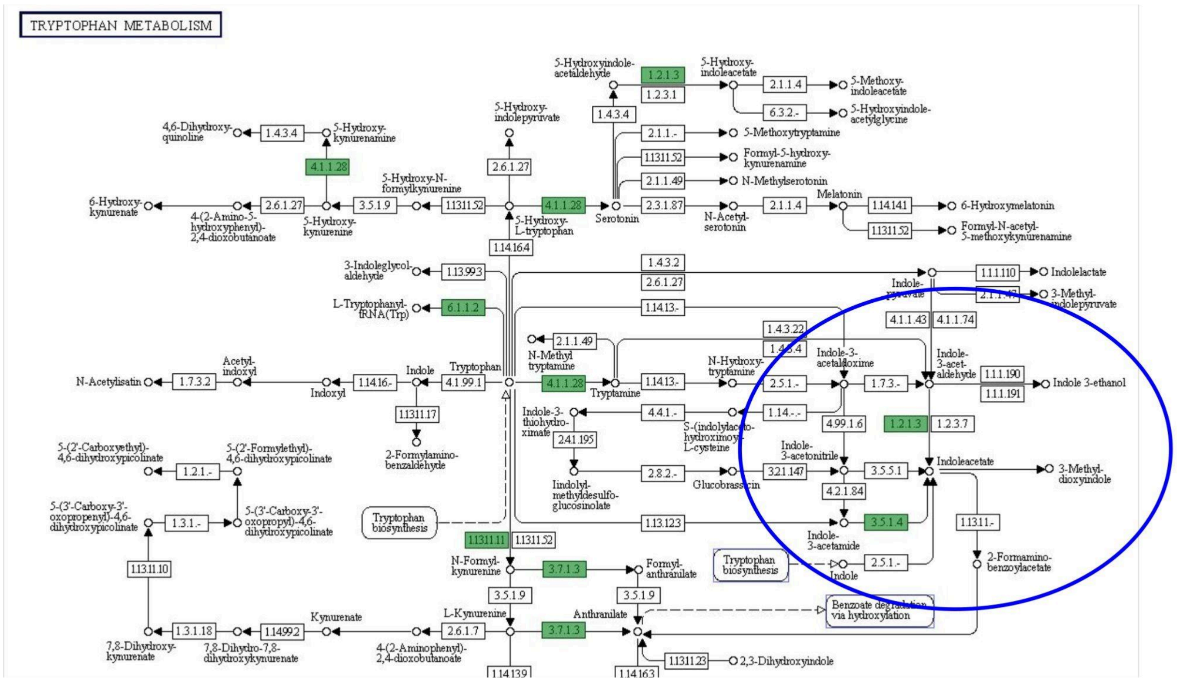

Figure S8: *Indole-3-acetic acid biosynthesis Pathway (highlighted in blue circle). The IAA synthesis, here green color highlighted boxes indicate the presence of genes in the Amycolatopsis* sp. BCA-696 *assembly (based on RAST annotation(generated from KEGG Database* https://www.kegg.jp*)).*

Supplementary Tables

Table S1: Summary of whole genome sequencing data before and after pre-processing of reads.

		Paired-end reads			Mate-pair reads		
	**Library**	**Average sequence Length (bp)**	**Number of sequences (millions)**	**Number of Bases (millions)**	**Average sequence length**	**Number of sequences (millions)**	**Number of Bases (millions)**
Raw reads	Forward	100	9.905	990.5	250	6.242	1560.4
	Reverse	100	9.905	990.5	250	6.242	1560.4
Pre-processed reads	Forward	91	9.568	867.2	174	4.553	791.2
	Reverse	90	9.568	859.1	167	4.553	761.9
	Unpaired (forward)	87	0.241	21.1	192	0.980	188.4
	Unpaired (reverse)	78	0.019	1.5	209	0.615	128.5

Table S2. Unique genes in *Amycolatopsis* sp. BCA-696 with known/predicted functions (from sources like RAST, KEGG, Reciprocal Best-BLAST (RBB) to reference bacterial genomes).

NCBI Protein ID	Start	Stop	Annotation from RAST				Annotation from KEGG	Annotation from Reciprocal Best BLAST
			Role	Category	Subcategory	Subsystem	Process /pathway	
LCL61_20900[#]	4455825	4456508	Alpha-1,2-mannosidase	Carbohydrates	Monosaccharides	Mannose Metabolism	Not Included in Pathway,typical type II membrane protein.	Analysis not carried out
WYW17692.1	4119790	4120041	Galactonate dehydratase (EC 4.2.1.6); Ontology_term	-	-	Muconate lactonizing enzyme family	ec00052 Galactose metabolism ec01100 Metabolic pathways ec01120 Microbial metabolism in diverse environments	Analysis not carried out
WYW20920.1	7667892	7668284	Cysteine desulfurase (EC 2.8.1.7); Ontology_term	-	Plant-Prokaryote DOE project	Iron-sulfur cluster assembly	map00730 Thiamine metabolism map01100 Metabolic pathways map01240 Biosynthesis of cofactors map04122 Sulfur relay system	Analysis not carried out
LCL61_37625[#]	8057564	8058400	Membrane alanine aminopeptidase N (EC 3.4.11.2); Ontology_term	Protein Metabolism	Protein degradation	Aminopeptidases (EC 3.4.11.-)	map00480 Glutathione metabolism map01100 Metabolic pathways	Analysis not carried out
WYW17944.1	4371834	4371974	Quinone oxidoreductase (EC 1.6.5.5); Ontology_term	Respiration	-	Quinone oxidoreductase family	Not Included in Pathway or Brite	Analysis not carried out
WYW21370.1	8163572	8164237	diguanylate cyclase/phosphodiesterase (GGDEF & EAL domains) with PAS/PAC sensor(s)	Stress Response	-	Bacterial hemoglobins	Not Included in Pathway or Brite	Analysis not carried out
LCL61_37735[#]	8081570	8081698	S-methyl-5-thioribose-1-pho	-	-	-	map00270 Cysteine and	Analysis not carried out

			sphate isomerase (EC 5.3.1.23);Ontology_term				methionine metabolism map01100 Metabolic pathways	
WYW18527.1	5039270	5039431	Carboxyvinyl-carboxyphosphonate phosphorylmutase (EC 2.7.8.23); Ontology_term	-	-	-	map00440 Phosphonate and phosphinate metabolism map01110 Biosynthesis of secondary metabolites	Analysis not carried out
WYW19642.1	6267796	6269049	xylosidase/arabinosidase	-	-	-	map00520 Amino sugar and nucleotide sugar metabolism map01100 Metabolic pathways	Analysis not carried out
WYW14042.1	169245	169391	FIG00997878: hypothetical protein	-	-	-		No annotation available
WYW15294.1	1542101	1542613	Cell division-associated ATP-dependent zinc metalloprotease FtsH	-	-	-	-	No annotation available
WYW15886.1	2146194	2146997	Oxidoreductase short-chain dehydrogenase/reductase family	-	-	-	-	No annotation available
WYW13939.1	4162352	4162471	probable ABC drug resistance transporter permease component	-	-	-	-	No annotation available
fig|1813.56.peg.4047*	4215642	4216130	Inner-membrane proton/drug antiporter (MSF type) of tripartite multidrug efflux system	-	-	-	-	No annotation available
WYW18175.1	4651710	4652408	FIG022780: hypothetical protein	-	-	-	-	No annotation available
WYW18798.1	5336030	5336377	Transcriptional regulator, MarR family	-	-	-	-	No annotation available
WYW18814.1	5359321	5359725	Acetyltransferase, GNAT	-	-	-	-	No annotation available

			family					
WYW19316.1	5899136	5899285	Transcriptional regulator, GntR family	-	-	-	-	No annotation available
LCL61_28240[#]	6027666	6027791	CRISPR-associated protein Cas2	-	-	-	-	No annotation available
WYW19441.1	6027920	6028741	CRISPR-associated protein Cas1	-	-	-	-	No annotation available
WYW19496.1	6093791	6095110	KtrAB potassium uptake system, integral membrane component KtrB	-	-	-	-	No annotation available
WYW19666.1	6297868	6298518	Putative protein-S-isoprenylcysteine methyltransferase	-	-	-	-	No annotation available
WYW19670.1	6300040	6301503	ABC transporter, permease protein	-	-	-	-	No annotation available
fig|1813.56.peg.6987*	7328269	7328379	FIG01136066: hypothetical protein	-	-	-	-	No annotation available
WYW13995.1	103674	105323	periplasmic protein	-	-	-	-	No annotation available
WYW14288.1	8090795	8091028	short-chain dehydrogenase/oxidoreductase	-	-	-	-	No annotation available
LCL61_40725[#]	8701829	8702200	Uncharacterized MFS-type transporter	-	-	-	-	No annotation available
fig|1813.56.peg.8470*	8821199	8821327	hypothetical glycine-rich protein	-	-	-	-	No annotation available
WYW15371.1	1617656	1618411	hypothetical protein	-	-	-	-	WP_123099137.1 MAE_28990/MAE_18760 family HEPN-like nuclease [Streptomyces

								botrytidirepellens]
WYW13928.1	447	692	hypothetical protein	-	-	-	-	WP_233439918.1 sigma-70 family RNA polymerase sigma factor [Lentzea atacamensis]
fig|1813.56.peg.2418*	2521102	2521335	hypothetical protein	-	-	-	-	WP_089955919.1 STAS domain-containing protein [Lentzea xinjiangensis]
WYW16258.1	2521456	2521695	hypothetical protein	-	-	-	-	WP_130477081.1 NaeI family type II restriction endonuclease, [Amycolatopsis suaedae],CCH32891.1 Putatibe restriction endonuclease [Saccharothrix espanaensis DSM 44229]
WYW16261.1	2523624	2524280	hypothetical protein	-	-	-	-	WP_139239184.1 HNH endonuclease [Actinopolymorpha cephalotaxi]
WYW16263.1	2529196	2530779	hypothetical protein	-	-	-	-	MBP2323042.1 NAD(P)-dependent dehydrogenase (short-subunit alcohol dehydrogenase family) [Kibdelosporangium banguiense]
WYW16307.1	2569547	2570737	hypothetical protein	-	-	-	-	AGO97172.1 transport protein [Streptomyces sp. CNT-179], BCW42699.1 sugar ABC transporter substrate-binding protein [Arthrobacter sp. StoSoilB3]
WYW17366.1	3661384	3661932	hypothetical protein	-	-	-	-	WP_254752965.1 chloramphenicol phosphotransferase CPT family protein [Streptomyces sp. NEAU-Y11], WP_148589949.1 guanylate kinase [Streptomyces sp. WAC01526]
LCL61_17985#	3747075	3747203	hypothetical protein	-	-	-	-	SER99179.1 Transposase DDE domain-containing protein [Lentzea xinjiangensis]
LCL61_02025#	471423	471617	hypothetical protein	-	-	-	-	WP_187280630.1 transposase, partial

								[Microbispora sp. CSR-4]
WYW18189.1	4667916	4668542	hypothetical protein	-	-	-	-	WP_242382180.1 L-2-amino-thiazoline-4-carboxylic acid hydrolase [Actinomadura sp. ATCC 31491]
WYW18587.1	5107740	5108024	hypothetical protein	-	-	-	-	WP_031510787.1 DUF1540 domain-containing protein [Streptomyces megasporus]
WYW18707.1	5245827	5246171	hypothetical protein	-	-	-	-	WP_037273706.1 DUF2267 domain-containing protein [Kibdelosporangium aridum]
fig|1813.56.peg.5095*	5336551	5336736	hypothetical protein	-	-	-	-	EFL06535.1 transposase [Streptomyces sp. AA4]
WYW18821.1	5366846	5367985	hypothetical protein	-	-	-	-	WP_206807638.1 DDE-type integrase/transposase/recombinase [Amycolatopsis sp. 195334CR], MBN1174856.1 Mu transposase C-terminal domain-containing protein [Micromonosporaceae bacterium]
WYW19401.1	5991134	5991952	hypothetical protein	-	-	-	-	WP_209639684.1 recombinase family protein [Kibdelosporangium banguiense]
LCL61_28240[#]	6027381	6027626	hypothetical protein	-	-	-	-	WP_246459111.1 type I-E CRISPR-associated endoribonuclease Cas2e [Amycolatopsis jiangsuensis]
WYW19483.1	6082570	6082806	hypothetical protein	-	-	-	-	WP_009151800.1 universal stress protein [Saccharomonospora marina]
WYW19539.1	6140644	6141483	hypothetical protein	-	-	-	-	WP_121005597.1 nucleotidyltransferase domain-containing protein [Saccharothrix australiensis], QTR02959.1 DUF4111 domain-containing protein [Saccharothrix algeriensis]

WYW19546.1	6147801	6148976	hypothetical protein	-	-	-	-	WP_025349737.1 sterol carrier protein domain-containing protein [Nocardia nova], WP_201843379.1 GNAT family N-acetyltransferase [Streptomyces actinomycinicus]
WYW19549.1	6150698	6152221	hypothetical protein	-	-	-	-	WP_053701929.1 Gfo/Idh/MocA family oxidoreductase [Streptomyces sp. WM6368]
WYW19550.1	6152218	6153228	hypothetical protein	-	-	-	-	WP_032755484.1 MULTISPECIES: phosphotransferase [Streptomyces microflavus subgroup] streptomycin phosphotransferase (aphE) gene
WYW20560.1	7300000	7300188	hypothetical protein	-	-	-	-	WP_204032210.1 ATP-binding protein [Micromonospora qiuiae]
WYW20957.1	7706325	7707278	hypothetical protein	-	-	-	-	WP_165965202.1 GIY-YIG nuclease family protein [Actinomadura bangladeshensis],CNG13537.1 T5orf172 domain [Mycobacterium tuberculosis]

# indicates that the RAST IDs were mapped to a pseudo gene, hence only the Locus tag in NCBI protein annotation has been provided.

* indicates that the RAST IDs did not map to any proteins annotated by NCBI, so the RAST IDs themselves were provided.

Table S3. Prediction of Biosynthetic gene clusters (BGCs) and their domain/class in *Amycolatopsis* sp. BCA-696 genome using PRISM4 tool. Further insights into the role of core and additional biosynthetic genes were deduced using a homology search between their sequences to KEGG ortholog/pathway database, and hits were recorded under the column "Associated KEGG pathway".

Cluster No	Start	End	Strand	BGC Domain	BGC Class	Associated KEGG pathway (gene ID)
Cluster 1	28530	29427	+	Glycopeptide Resistance: D-lactate dehydrogenase	Resistance	(Not searched)
	29428	30469	+	Glycopeptide: D-ala-D-lactate ligase	Resistance	(Not searched)
	30465	31074	+	Glycopeptide resistance: D-Ala-D-Ala dipeptidases	Resistance	(Not searched)
	33381	34221	+	Prephenate dehydrogenase	Aminocoumarin	(Not searched)
	36435	42621	+	Adenylation,Thiolation,Condensation, Epimerization	Thiotemplated	Biosynthesis of vancomycin group antibiotics (CepA)
	42689	45773	+	Adenylation	Thiotemplated	(Not searched)
	46494	58494	+	Adenylation	Thiotemplated	Biosynthesis of vancomycin group antibiotics (CepB)
	58633	64195	+	Adenylation	Thiotemplated	Biosynthesis of vancomycin group antibiotics (CepC)
	64646	65822	+	P450A	Tailoring	Biosynthesis of vancomycin group antibiotics (CepE)
	65845	67003	+	P450D	Tailoring	Biosynthesis of vancomycin group antibiotics (CepE)
	66992	68189	+	P450B	Tailoring	Biosynthesis of vancomycin group antibiotics (CepF)
	68430	69672	+	P450C	Tailoring	Biosynthesis of vancomycin group antibiotics (CepG)
	69684	70842	+	Glycosyltransferase	Tailoring	Biosynthesis of vancomycin group antibiotics (GtfD)
	70874	72092	+	Glycosyltransferase	Tailoring	Biosynthesis of vancomycin group antibiotics (GtfD)
	72108	73323	+	Glycosyltransferase	Tailoring	(Not searched)
	74191	75319	+	Glycosyltransferase	Tailoring	(Not searched)
	76312	78109	+	Glycosyltransferase	Tailoring	(Not searched)
	79455	80759	+	Aminotransferase		Biosynthesis of vancomycin group antibiotics (HpgT)
	80918	81743	+	Thioesterase	Thiotemplated	Biosynthesis of vancomycin group antibiotics (CepJ)
	81739	83476	+	Adenylation	Thiotemplated	Biosynthesis of vancomycin group antibiotics (CepK)

	83491	84681	+	P450	Tailoring	Biosynthesis of vancomycin group antibiotics (CepL)
	84784	85845	+	4-hydroxyphenylpyruvate dioxygenase		Biosynthesis of vancomycin group antibiotics (HmaS)
	85842	86915	+	L-lactate dehydrogenase		Biosynthesis of vancomycin group antibiotics (Hmo)
	87272	88682	+	2,3-dehydratase	Deoxysugar	Biosynthesis of vancomycin group antibiotics (EvaA)
	88683	89649	+	4-ketoreductase	Deoxysugar	Biosynthesis of vancomycin group antibiotics (VcaE)
	89645	90755	+	3-aminotransferase	Deoxysugar	Biosynthesis of vancomycin group antibiotics (EvaB)
	90771	91389	+	Epimerase	Deoxysugar	Biosynthesis of vancomycin group antibiotics (EvaC)
	93609	94709	+	Polyketide synthase (PKS)	Type III polyketide	Biosynthesis of vancomycin group antibiotics (dpgA, dpgB, dpgD)
	94703	95365	+			(Not searched)
	95362	96579	+			Biosynthesis of vancomycin group antibiotics (dpgC)
Cluster2	646201	646510	-	Anthrone-type oxygenase	Type II polyketide	(Not searched)
	649559	650342	+	Thioesterase	Thiotemplated	(Not searched)
	652326	653013	+	Cyclodipeptide synthase	Cyclodipeptide	(Not searched)
Cluster 3	823934	824480	-	Linaridin precursor (legonaridin-type)	RiPP	(Not searched)
	832999	833170	+	Dehydratase	Thiotemplated	(Not searched)
	833635	834862	-	Glycosyltransferase	Tailoring	(Not searched)
	837879	839619	+	Acyl adenylating enzyme	Thiotemplated	(Not searched)
	839615	843812	+	Thiolation,Ketosynthase,Acyltransferase,Thiolation,Thioesterase	Thiotemplated	(Not searched)
Cluster 4	877649	878780	-	Glycosyltransferase	Tailoring	(Not searched)
	878776	879553	-	Thioesterase	Thiotemplated	(Not searched)
	879566	879866	-	Thiolation	Thiotemplated	(Not searched)
	879882	883902	-	Ketosynthase	Thiotemplated	(Not searched)
	883898	890210	-	Ketosynthase,Acyltransferase,Thiolation,Ketoreductase	Thiotemplated	(Not searched)
Cluster 5	1952898	1953288	+	Ectoine synthase	Ectoine	(Not searched)
Cluster 6	2381161	2383714	+	Lantipeptide kinase/cyclase (LanKC or LanL)	RiPP	(Not searched)
	2383754	2383898	+	Lantipeptide precursor (clade H1)	RiPP	(Not searched)
Cluster 7	3195798	3197049	-	Ketosynthase	Thiotemplated	(Not searched)
	3197089	3197335	-	Thiolation	Thiotemplated	(Not searched)
	3198383	3199262	-	Acyltransferase	Thiotemplated	(Not searched)

Cluster 8	3372806	3373750	+			Biosynthesis of Enediyne antibiotics (SgcE3)
	3373747	337511	+			Biosynthesis of Enediyne antibiotics (SgcE4)
	3375717	3376703	+			Biosynthesis of Enediyne antibiotics (SgcE5)
	3376700	3378052	+			Biosynthesis of Enediyne antibiotics (SgcE7)
	3378058	3378615	+			Biosynthesis of Enediyne antibiotics (SgcE8)
	3378612	3380279	+			Biosynthesis of Enediyne antibiotics (SgcE9)
	3380282	3381205		alkylhydroperoxidase like protein%2C AhpD family		(Not searched)
	3382009	3387838	+	Ketosynthase,Acyltransferase,Ketoreductase, Enediyne C-terminal phosphopantetheinyltransferase	Thiotemplated	Biosynthesis of Enediyne antibiotics (SgcE)
	3387840	3388280	+			Biosynthesis of Enediyne antibiotics (SgcE10)
	3388282	3388854	+			Biosynthesis of Enediyne antibiotics (MapE6)
Cluster 9	3686900	3689348	-	Nitroreductase	Thiotemplated	No hit
	3695032	3696006				Biosynthesis of Enediyne antibiotics (CalO4)
	3696056	3701006	-	Ketosynthase, Acyltransferase, Ketoreductase, Thiolation	Thiotemplated	Biosynthesis of Enediyne antibiotics (mdpB)
	3701198	3702287	-	Glycosyltransferase	Tailoring	(Not searched)
	3702492	3703350	+	Thioesterase	Thiotemplated	(Not searched)
Cluster 10	3860828	3862102	-	3-dehydroquinate synthase		Acarbose and validamycin biosynthesis (4.2.3.152)
	3864919	3865738	+	Tyrosine ortho-hydroxylase	Lincoside	(Not searched)
	3870662	3871961	+	Acyltransferase, Thiolation	Thiotemplated	(Not searched)
	3871957	3877534	+	Ketosynthase, Dehydratase	Thiotemplated	(Not searched)
Cluster 11	3951971	3952771	-			No hit
	3952947	3954140	-			No hit
	3954845	3960062	-	Ketosynthase,Acyltransferase,Ketoreductase,Thiolation	Thiotemplated	Biosynthesis of Enediyne antibiotics (mdpB)

	3961583	3962879	-	Ketosynthase	Thiotemplated	(Not searched)
	3963018	3964992	+	Amidotransferase	Type II polyketide	(Not searched)
	3968606	3976322	-	Acyl adenylating enzyme,Thiolation,Ketosynthase,Acyl transferase,Dehydratase,Ketoreductase,Thiolation,Thioesteras	Thiotemplated	No hit
	3976346	3977837	-	Adenylation	Thiotemplated	(Not searched)
	3979037	3980030	+	Nitroreductase	Thiotemplated	(Not searched)
	3987984	3988938	+	Nitroreductase,Nitroreductase	Thiotemplated	(Not searched)
	3991157	3993680	+	Thiolation	Thiotemplated	No hit
	3993715	3996235	+	Adenylation,Thiolation	Thiotemplated	(Not searched)
	3997251	3998337	+	2,3-diaminopropionate biosynthesis protein SbnB	Beta-lactam	(Not searched)
	3998375	3999362	+	2,3-diaminopropionate biosynthesis protein SbnA	Beta-lactam	(Not searched)
Cluster 12	4512489	4513308	-	Thioesterase	Thiotemplated	(Not searched)
	4519960	4523389	+	Adenylation,Thiolation,Ketosynthase	Thiotemplated	(Not searched)
	4523385	4526844	+	Adenylation,Thiolation,Condensation,Thiolation	Thiotemplated	No hit
	4526854	4532185	+	Ketosynthase,Acyltransferase,Ketoreductase,Dehydratase,Thiolation	Thiotemplated	No hit
	4532509	4535536	+	Condensation,Adenylation,Thiolation	Thiotemplated	(Not searched)
	4535532	4540962	+	Condensation,Adenylation,Thiolation,Thioesterase	Thiotemplated	No hit
Cluster 13	5086732	5086909	-	Putative lantipeptide precursor	RiPP	(Not searched)
	5090326	5093152	-	Lantipeptide fused dehydratase/cyclase	RiPP	(Not searched)
	5102686	5103151	+	Thioesterase	Thiotemplated	(Not searched)
Cluster 14	5340989	5342567	-	Adenylation	Thiotemplated	(Not searched)
	5342563	5343223	-	Piperazic acid synthase	Rare monomer biosynthesis	(Not searched)
	5347520	5348723	+	P450C	Tailoring	(Not searched)
	5348917	5352658	-	Condensation,Adenylation,Thioesterase	Thiotemplated	(Not searched)
	5352657	5354511	-	Amidotransferase	Type II polyketide	(Not searched)
	5354553	5356107	-	Glutamine synthetase homologue - Acetylhydrazine transferase	Phosphonate	(Not searched)
	5356116	5357535	-	Acetylhydrazinearginine deacetylase	Phosphonate	(Not searched)
	5359320	5359725	-	Hydrazinosuccinate N-acetyltransferase	Phosphonate	(Not searched)
	5359711	5361016	-	Acetylhydrazinosuccinate lyase	Phosphonate	(Not searched)
	5361036	5363085	-	Condensation	Thiotemplated	(Not searched)
	5363117	5363375	-	Thiolation	Thiotemplated	(Not searched)
	5368726	5369632	-	Enoylreductase	Thiotemplated	(Not searched)
	5374217	5374823	-	Thioesterase	Thiotemplated	(Not searched)
Cluster 15	5611746	5633991	-	Condensation,Adenylation,Thiolation,	Thiotemplated	No hit
	5635515	5636463	+	Formyltransferase	Tailoring	(Not searched)
	5642604	5644242	-	Hygromycin ABC transporter	Resistance	(Not searched)

Cluster 16	5752830	5754237	-	EmrB: drug resistance MFS transporter	Resistance	(Not searched)
	5754233	5764190	-	Condensation,Adenylation,Thiolation,E	Thiotemplated	No hit
	5768857	5769091	-	Thiolation	Thiotemplated	(Not searched)
	5769087	5769753	-	Phenazine biosynthesis isochorismatase	Other	(Not searched)
	5769755	5771378	-	Acyl adenylating enzyme	Thiotemplated	(Not searched)
	5776593	5777172	+	D-Ala-D-Ala carboxypeptidase	Resistance	(Not searched)
	5781676	5783092	-	Adenylation	Thiotemplated	(Not searched)
Cluster 17	5904000	5910423	-	Thiolation,Ketosynthase,Acyltransferase,Dehydratase,Ketoreductase,Thiolation,Thioesterase	Thiotemplated	(Not searched)
	5910419	5912180	-	Acyl adenylating enzyme	Thiotemplated	(Not searched)
	5917646	5918540	+	Beta lactamase class A	Resistance	(Not searched)
	5923836	5924625	-	Thioesterase	Thiotemplated	(Not searched)
	5925184	5926108	-	Thioesterase	Thiotemplated	(Not searched)
Cluster 18	6196743	6197046	-	Anthrone-type oxygenase	Type II polyketide	(Not searched)
	6197386	6198139	-	C19 ketoreductase	Type II polyketide	(Not searched)
	6198663	6199197	-	Type II polyketide cyclase, clade 7 (tetracenomycins/pentangular polyphenols, 1st/2nd/3rd rings)	Type II polyketide	(Not searched)
	6199198	6199486	-	Thiolation	Thiotemplated	(Not searched)
	6199482	6200733	-	Chain length factor (KSβ)	Type II polyketide	(Not searched)
	6204418	6204754	+	Type II polyketide cyclase, clade 5a (pentangular polyphenols, 4th/5th rings)	Type II polyketide	(Not searched)
	6204750	6205179	+	Type II polyketide cyclase, clade 1 (tetracenomycins/pentangular polyphenols,	Type II polyketide	(Not searched)
	6205175	6206429	+	Ketosynthase α	Type II polyketide	(Not searched)
	6207033	6209007	+	Thiolation,Condensation,Thiolation	Thiotemplated	(Not searched)
	6211766	6214205	-	N-methyltransferase,C-methyltransferase	Deoxysugar	(Not searched)
	6214201	6220513	-	Ketosynthase,Acyltransferase,Ketoreductase,Ketoreductase,Thiolation,Condensation,Thioesterase	Thiotemplated	(Not searched)
	6220509	6224319	-	Thiolation,Condensation,Adenylation,Thiolation	Thiotemplated	No hit found
	6228935	6230360	+	Multidrug MFS transporter	Resistance	(Not searched)
	6236266	6237553	+	Thiolation	Thiotemplated	(Not searched)
Cluster 19	6228935	6230360	+	Multidrug MFS transporter	Resistance	(Not searched)
	6236266	6237553	+	Thiolation	Thiotemplated	(Not searched)
	6237549	6240579	+	Ketosynthase	Thiotemplated	(Not searched)
	6248095	6248380	-	Thiolation	Thiotemplated	(Not searched)
	6248403	6250125	-	Acyl adenylating enzyme	Thiotemplated	(Not searched)

	6251392	6253081	-	Carbamoyltransferase	Tailoring	(Not searched)
Cluster 20	6583022	6583781	-	Phosphopantetheinyltransferase	Thiotemplated	(Not searched)
	6583777	6584524	-	Thioesterase	Thiotemplated	(Not searched)
	6585566	6586706	-	Acyl-CoA dehydrogenase (methoxymalonate biosynthesis)	Rare monomer biosynthesis	(Not searched)
	6586719	6586971	-	Thiolation	Thiotemplated	(Not searched)
	6587919	6590850	-	Ketosynthase,Acyltransferase	Thiotemplated	(Not searched)
	6590858	6599591	-	Ketosynthase,Ketosynthase,Ketoreductase,Thiolation,Ketosynthase,Ketoreductase,Ketosynthase	Thiotemplated	(Not searched)
	6599799	6610794	-	Ketosynthase,Acyltransferase,Dehydratase,	Thiotemplated	(Not searched)
	6610827	6632565	-	Ketosynthase,Acyltransferase,Dehydratase,	Thiotemplated	(Not searched)
	6632589	6636924	-	Ketosynthase,Acyltransferase,Ketoreductase,Thiolation	Thiotemplated	(Not searched)
	6636923	6644648	-	Ketosynthase,Acyltransferase,Dehydratase,Ketoreductase,Thiolation,Thiolation,Acyltransferase	Thiotemplated	(Not searched)
	6644887	6651343	-	Thiolation,Ketosynthase,Acyltransferase,Dehydratase,Enoylreductase,Ketoreductase,Ketoreductase	Thiotemplated	(Not searched)
	6651648	6653340	+	Arginine monooxygenase	Tailoring	(Not searched)
	6653359	6654772	+	4-guanidinylbutanoate:CoA ligase	Tailoring	(Not searched)
	6654799	6655738	+	4-guanidinylbutanoyl-CoA:ACP acyltransferase	Tailoring	(Not searched)
	6655734	6657279	+	Multidrug MFS transporter	Resistance	(Not searched)
Cluster 21	7604708	7604972	+	Thiolation	Thiotemplated	(Not searched)
	7607376	7609323	+	Adenylation	Thiotemplated	(Not searched)
	7609334	7614092	+	Condensation,Condensation,Adenylation,Thiolation,Reductase	Thiotemplated	Myxalamid S biosynthesis (MxaA), a myxobacterial product
	7610988	7611054	-	Dehydratase	Thiotemplated	(Not searched)
Cluster 22	8006702	8007131	-	Lasso peptide transglutaminase homolog	RiPP	(Not searched)
	8007378	8009223	-	Lasso peptide asparagine synthase homolog	RiPP	(Not searched)
	8009244	8009373	-	Lasso peptide precursor	RiPP	(Not searched)
Cluster 23	8020295	8021375	+	KasQ 2-epimerase	Aminoglycoside	(Not searched)
	8026126	8027875	+	Acyl adenylating enzyme	Thiotemplated	(Not searched)
	8031408	8031657	+	Thiolation	Thiotemplated	(Not searched)
	8031653	8034602	+	Ketosynthase,Acyltransferase,Thiolation	Thiotemplated	(Not searched)
	8034615	8035641	+	ACP-glycerate acyltransferase and ketosynthase	Tailoring	(Not searched)
	8035637	8037572	+	1,3-Bisphospho-Glycerate S-acyltransferase and phosphatase	Tailoring	(Not searched)
	8037568	8037796	+	Thiolation	Thiotemplated	(Not searched)

	8037792	8038563	+	Two-component tetronate/tetramate Dieckmann cyclase/acetyltransferase	Tailoring	(Not searched)
	8038562	8039615	+	Two-component tetronate/tetramate Dieckmann cyclase/acetyltransferase	Tailoring	(Not searched)

Table S4. Genes annotated to 'siderophore' sub-system in RAST annotation.

NCBI Protein ID	Type	Start	Stop	Strand	Function
WYW14728.1	CDS	912638	913186	+	Isochorismatase (EC 3.3.2.1)
WYW15333.1	CDS	1582984	1584015	+	ABC-type Fe3+-siderophore transport system, permease component
WYW15334.1	CDS	1584012	1585016	+	ABC-type Fe3+-siderophore transport system, permease 2 component
WYW15977.1	CDS	2235468	2236607	+	Isochorismate synthase (EC 5.4.4.2) @ Isochorismate synthase (EC 5.4.4.2) of siderophore biosynthesis
WYW16309.1	CDS	2572150	2572914	-	Thioesterase in siderophore biosynthesis gene cluster
WYW17509.1	CDS	3882650	3883909	-	Salicylate synthetase (EC 5.4.4.2)(EC 4.2.99.21) @ Salicylate synthetase (EC 5.4.4.2)(EC 4.2.99.21) of siderophore biosynthesis
WYW17566.1	CDS	3997252	3998337	+	2,3-diaminopropionate for siderophore biosynthesis protein SbnB
WYW17567.1	CDS	3998376	3999362	+	2,3-diaminopropionate for siderophore biosynthesis protein SbnA
WYW18804.1	CDS	5343236	5344582	-	Siderophore biosynthesis protein, monooxygenase
WYW19070.1	CDS	5603323	5604360	-	ABC-type Fe3+-siderophore transport system, permease 2 component
WYW19071.1	CDS	5604384	5605427	-	ABC-type Fe3+-siderophore transport system, permease component
WYW19077.1	CDS	5611747	5633991	-	Siderophore biosynthesis non-ribosomal peptide synthetase modules
WYW19078.1	CDS	5634035	5635378	-	Siderophore biosynthesis protein, monooxygenase
WYW19195.1	CDS	5764184	5764960	-	ABC-type Fe3+-siderophore transport system, ATPase component
WYW19196.1	CDS	5764957	5765997	-	ABC-type Fe3+-siderophore transport system, permease component
WYW19197.1	CDS	5765994	5766977	-	putative siderophore transport system permease protein
WYW19199.1	CDS	5768007	5768861	-	Iron-chelator utilization protein
WYW19200.1	CDS	5768858	5769091	-	Isochorismatase of siderophore biosynthesis # fragment
WYW19201.1	CDS	5769088	5769753	-	Isochorismatase (EC 3.3.2.1) of siderophore biosynthesis
WYW19202.1	CDS	5769756	5771378	-	2,3-dihydroxybenzoate-AMP ligase (EC 2.7.7.58) of siderophore biosynthesis
WYW19203.1	CDS	5771375	5772571	-	Isochorismate synthase (EC 5.4.4.2) @ Isochorismate synthase (EC 5.4.4.2) of siderophore biosynthesis
WYW19204.1	CDS	5772721	5773470	-	2,3-dihydro-2,3-dihydroxybenzoate dehydrogenase (EC 1.3.1.28) of siderophore biosynthesis
WYW19605.1	CDS	6207034	6209007	+	Siderophore biosynthesis non-ribosomal peptide synthetase modules
WYW19606.1	CDS	6209004	6210077	+	Putative reductoisomerase in siderophore biosynthesis gene cluster
WYW19737.1	CDS	6359202	6359756	-	Isochorismatase (EC 3.3.2.1)
WYW19952.1	CDS	6583778	6584524	-	Thioesterase in siderophore biosynthesis gene cluster
WYW20353.1	CDS	7080190	7081017	+	ABC-type Fe3+-siderophore transport system, ATPase component
WYW20810.1	CDS	7552770	7553330	+	Isochorismatase (EC 3.3.2.1)
WYW21244.1	CDS	8011182	8011658	-	Poly-gamma-glutamate synthase subunit PgsC/CapC (EC 6.3.2.-)
WYW21245.1	CDS	8011662	8012885	-	Poly-gamma-glutamate synthase subunit PgsB/CapB (EC 6.3.2.-)
WYW21333.1	CDS	8122538	8123353	+	Ferric hydroxamate ABC transporter (TC 3.A.1.14.3), ATP-binding protein FhuC

WYW21334.1	CDS	8123371	8124348	+	Ferric hydroxamate ABC transporter (TC 3.A.1.14.3), periplasmic substrate binding protein FhuD
WYW21335.1	CDS	8124348	8126387	+	Ferric hydroxamate ABC transporter (TC 3.A.1.14.3), permease component FhuB
WYW21863.1	CDS	8686544	8687890	+	Salicylate synthetase (EC 5.4.4.2)(EC 4.2.99.21) @ Salicylate synthetase (EC 5.4.4.2)(EC 4.2.99.21) of siderophore biosynthesis
WYW21865.1	CDS	8688932	8690560	+	2,3-dihydroxybenzoate-AMP ligase (EC 2.7.7.58) of siderophore biosynthesis
WYW21927.1	CDS	8751903	8752697	-	ABC-type Fe3+-siderophore transport system, ATPase component
WYW21928.1	CDS	8752694	8753752	-	ABC-type Fe3+-siderophore transport system, permease 2 component
WYW21929.1	CDS	8753749	8754756	-	ABC-type Fe3+-siderophore transport system, permease component

Table S5. Siderophore biosynthesis genes in *Amycolatopsis sp.* BCA-696 predicted using Reciprocal BLAST.

**S. no**	**Siderophore biosynthesis step**	**Enzyme name/description**	**Genes predicted by RAST**	**Genes predicted by Reciprocal BLAST** (Reference gene name, ID, and species)
1	Intermediate	Isochorismate synthase [EC:5.4.4.2]	WYW19203.1	(Not required)
2	Intermediate	Isochorismatase [EC:3.3.2.1]	WYW19200.1, WYW19201.1	(Not required)
3	Intermediate	2,3-dihydro-2,3-dihydroxybenzoate dehydrogenase [EC:1.3.1.28]	WYW19204.1	(Not required)
4	Enterobactin	Enterobactin synthetase component D (entD) [EC:6.3.2.14, EC:2.7.8.-]	(annotation not found)	No ortholog found (entD; NP_415115; *E. coli*)
5	Enterobactin	L-serine---[L-seryl-carrier protein] ligase (EntF) [EC:6.3.2.14, EC:6.2.1.72]	(annotation not found)	WYW13957.1, WYW13959.1, WYW18072.1, WYW18073.1, WYW18809.1, WYW13975.1, WYW18809.1 (entF; NP_415118.1; *E.coli*)
6	Enterobactin, Bacillibactin, Myxochelin, Vibriobactin	2,3-dihydroxybenzoate---[aryl-carrier protein] ligase (entE, vibE, dhbE, mxcE) [EC:6.3.2.14, EC:6.2.1.71]	(annotation not found)	WYW19202.1, WYW21865.1 (entE; NP_415126; *E.coli*)
7	Enterobactin, Bacillibactin, Myxochelin, Vibriobactin	bifunctional isochorismate lyase / aryl carrier protein (entB, dhbB, vibB, mxcF) [EC:6.3.2.14, EC:3.3.2.1]	WYW19201.1	(not required)
8	Bacillibactin	glyine---[glycyl-carrier protein] ligase (dhbF) [EC:6.2.1.66]	(annotation not found)	No ortholog found (dhbF; NP_391076; *B. subtilis*)
9	Myxochelin	nonribosomal peptide synthetase (mxcG)	(annotation not found)	WYW16323.1, WYW16323.1, WYW13958.1, WYW13959.1, WYW18070.1, WYW18072.1, WYW18073.1, WYW18683.1, WYW18809.1, WYW19194.1, WYW13975.1, WYW20866.1, WYW20867.1 (mxcG; AYC38396/ WP_120050897; *S. griseorubiginosu*s)
10	Vibriobactin	vibriobactin synthetase (vibH)	(annotation not found)	No ortholog found (vibH; ABQ20274/WP_001880577; *V. cholerae* O395)
11	Vibriobactin	nonribosomal peptide synthetase (VibF)	(annotation not found)	No ortholog found

				(vibF; ABQ21224WP_000523394; *V. cholerae* O395)
12	Intermediate	Salicylate biosynthesis protein (EC 4.2.99.21)	WYW17509.1, WYW21863.1	(Not required)
13	Pyochelin	pyochelin biosynthesis protein PchG	(annotation not found)	No ortholog found (pchG; NP_252914; *P. aeruginosa* PAO1)
14	Pyochelin	L-cysteine---[L-cysteinyl-carrier protein] ligase (PchF) [EC:6.2.1.69]	(annotation not found)	WYW19611.1 (pchF; NP_252915; *P. aeruginosa* PAO1)
15	Pyochelin	L-cysteine---[L-cysteinyl-carrier protein] ligase PchE [EC:6.2.1.69]	(annotation not found)	No ortholog found (pchE; NP_252916; *P. aeruginosa* PAO1)
16	Pyochelin	salicylate---[aryl-carrier protein] ligase (pchD) [EC:6.2.1.61]	(annotation not found)	WYW21865.1 (pchD; NP_252918; *P. aeruginosa* PAO1)
17	Mycobactin	mycobactin salicyl-AMP ligase (mbtA) [EC:6.3.2.-]	(annotation not found)	WYW19202.1, WYW21865.1 (mtbA; NP_216900; *M. tuberculosis* H37Rv)
18	Mycobactin	mycobactin phenyloxazoline synthetase (mbtB)	(annotation not found)	WYW13956.1, WYW13957.1, WYW17564.1, WYW13958.1, WYW13959.1, WYW18070.1, WYW18072.1, WYW18073.1, WYW18802.1, WYW18809.1, WYW19077.1, WYW19194.1, WYW13975.1, WYW19611.1, WYW20866.1, WYW20867.1 (mtbB; NP_216899; *M. tuberculosis* H37Rv)
19	Mycobactin	mycobactin peptide synthetase (MbtE)	(annotation not found)	No ortholog found (mtbE; NP_216896; *M. tuberculosis* H37Rv)
20	Mycobactin	mycobactin polyketide synthetase (MbtC)	(annotation not found)	fig|1813.56.peg.3772 (mtbC; NP_216898; *M. tuberculosi*s H37Rv)
21	Mycobactin	mycobactin polyketide synthetase MbtD	(annotation not found)	No ortholog found (mtbD; NP_216897; *M. tuberculosis* H37Rv)
22	Mycobactin	mycobactin peptide synthetase MbtF	(annotation not found)	WYW13956.1, WYW13957.1, WYW17564.1, WYW13958.1, WYW13959.1, WYW18070.1, WYW18072.1, WYW18073.1, WYW18802.1, WYW18809.1, WYW19077.1, WYW19194.1, WYW13975.1, WYW19611.1, WYW20866.1, WYW20867.1 (mtbF; NP_216895; *M. tuberculosis* H37Rv)
23	Mycobactin	mycobactin lysine-N-oxygenase	(annotation not found)	WYW18804.1, WYW19078.1, WYW19804.1 (mtbG; NP_216894; *M.tuberculosis*)
24	Yersiniabactin	yersiniabactin salicyl-AMP ligase (irp5) [EC:6.3.2.-]	(annotation not found)	WYW21865.1 (irp5; CAL20546 / WP_001088826; *Y. pestis* CO92)
25	Yersiniabactin	yersiniabactin nonribosomal peptide synthetase (irp2)	(annotation not found)	No ortholog found (irp2; CAL20550; Y. pestis CO92)
26	Yersiniabactin	yersiniabactin synthetase, thiazolinyl reductase component (irp3)	(annotation not found)	No ortholog found (irp3; CAL20548/WP_000982866; *Y. pestis* CO92)
27	Yersiniabactin	yersiniabactin nonribosomal peptide/polyketide synthase (irp1)	(annotation not found)	WYW18069.1, WYW18071.1, WYW19610.1, WYW19957.1, WYW19961.1, WYW21262.1, WYW14673.1 (irp1; CAL20549/WP_002212777; *Y. pestis* CO92)

Table S6. Cellulose degradation pathway genes in *Amycolatopsis sp.* BCA-696 identified using RAST and/or predicted using Reciprocal BLAST.

S. no	Cellulase related enzyme groups	Genes predicted by RAST	Genes predicted by Reciprocal BLAST (Reference gene ID and species)
1	EC 3.2.1.4; Endoglucanase (lyses non-covalent bonds in cellulose)	WYW14055.1 , WYW17289.1, WYW18627.1, WYW20946.1	(Not required)
2	EC 3.2.1.91; Cellulose-1,4-beta-cellobiosidase (exoglucanase lyses cellulose into cellobiose)	(annotation not found)	WYW16187.1; (WP_072961769.1, *Vibrio gazogenes*)
3	EC 3.2.1.21; beta-glucosidase (lyses cellobiose into D-glucose by breaking Beta-1,4 linkages)	WYW15213.1, WYW16213.1, WYW17468.1, WYW17772.1, WYW18406.1, WYW18474.1, WYW18732.1, WYW18733.1	(Not required)
4	EC 2.4.1.20; cellobiose phosphorylase	(annotation not found)	WYW14533.1; (WP_072955605.1, *Vibrio gazogenes*)

Table S7. Chitin degradation pathway genes in *Amycolatopsis sp.* BCA-696 identified using RAST and/or predicted using Reciprocal BLAST.

S. no	Chitin degradation pathway enzyme groups	Genes predicted by RAST	Genes predicted by Reciprocal BLAST (Reference gene ID and species)
1	EC 3.2.1.14; chitinase	WYW14092.1, WYW18461.1, WYW19090.1, WYW19982.1, WYW20247.1, WYW20702.1, WYW20703.1, WYW20704.1, WYW20718.1, WYW20155.1, WYW20946.1	(Not required)
2	EC 3.2.1.52 - beta-N-acetylglucosaminidase	WYW17579.1, WYW21353.1	(Not required)
3	EC 2.4.1.280; N,N'-diacetylchitobiose phosphorylase;(chbP)	(annotation not found)	WYW16675.1; (WP_010985887.1, *Streptomyces* multispecies)
4	EC 3.5.1.41; chitin deacetylase;	(annotation not found)	WYW16665.1; (WP_010988692.1, *Streptomyces* multispecies)
5	EC 3.2.1.132; chitosanase	(annotation not found)	WYW14410.1; (WP_010984783.1, *Streptomyces avermitilis*)
6	EC 3.2.1.165 exo-1,4-beta-D-glucosaminidase	(annotation not found)	WYW20217.1; (WP_010982661.1, *Streptomyces avermitilis*)

Table S8. Lipase genes in *Amycolatopsis sp.* BCA-696 predicted using RAST and/or Reciprocal BLAST.

S. no	Lipase related enzyme groups	Genes predicted by RAST	Genes predicted by Reciprocal BLAST (Reference gene name, ID, and species)
1	EC 3.1.1.3 lipase; triacylglycerol lipase	WYW14559.1, WYW14683.1, WYW15448.1, WYW15930.1, WYW17685.1, WYW17803.1, WYW21207.1	(Not required)

2	EC 3.1.1.34<br>diacylglycerol lipase;lipC	(annotation not found)	none |only found in Animals
3	EC 2.3.1.158<br>phospholipid:diacylglycerol acyltransferase	(annotation not found)	none |only found in Plants, Fungi, Protista
4	EC 2.3.1.20<br>Diacylglycerol O-acyltransferase	WYW14154.1, WYW16592.1, WYW17238.1	(Not required)

Table S9. Indole-3-acetic acid biosynthesis genes in *Amycolatopsis sp.* BCA-696 identified using RAST and predicted using Reciprocal BLAST.

S. no	IAA biosynthesis pathway	Reactions	Enzymes	Genes predicted by RAST	Genes predicted by Reciprocal BLAST (Reference gene ID and species)
1	Via Tryptamine	Tryptophan → Tryptamine	EC 4.1.1.28 (Trp decarboxylase)	WYW16295.1, WYW17444.1	(Not required)
2		Tryptamine → Indole 3-acetaldehyde	EC 1.4.3.22, 1.4.3.4 (Amine/Tyramine oxidase)	WYW19157.1	(Not required)
3		Indole 3-acetaldehyde→ Indole acetate	EC 1.2.1.3, 1.2.3.7 (Indole 3-acetaldehyde dehydrogenase)	WYW19157.1, WYW15227.1, WYW15494.1, WYW16353.1, WYW17439.1, WYW19175.1, WYW19367.1, WYW19620.1, WYW21802.1, WYW21803.1, WYW21925.1	(Not required)
4	Via Indole 3-pyruvate	Tryptophan → Indole 3-pyruvate	EC 2.6.1.99, 2.6.1.27, 1.4.3.2 (Aminotransferase)	(annotation not found)	(annotation not found)
5		Indole 3-pyruvate → Indole 3-acetaldehyde	EC 4.1.1.43, 4.1.1.74 (Indole pyruvate decarboxylase)	(annotation not found)	(annotation not found)
6		Indole 3-acetaldehyde→ Indole acetate	EC 1.2.1.3, 1.2.3.7 (Indole 3-acetaldehyde dehydrogenase)	(annotation not found)	WYW21802.1 (NCBI-ProteinID: ANN20607, *A. orientalis*)
7	Via Indole 3-acetamide	Tryptophan → Indole 3-acetamide	EC 1.13.12.3 (Tryptophan mono-oxygenase)	(annotation not found)	(annotation not found)
		Indole 3-acetamide → Indole acetate	EC 3.5.1.4 (IAM hydrolase)	WYW17127.1, WYW17851.1, WYW18812.1	(Not required)

Table S10. Details of seventy-six Amycolatopsis genomes, excluding BCA-696, with a scaffold or higher level assembly, were used for constructing the Pan-genome. The 14 closely related genomes of BCA-696, which included genomes with varying levels of assembly, included several from this list (highlighted in gray).

	Assembly	Assembly Name	Organism Name	Organism	Total	Assembly

	Accession			Infraspecific Names Strain	Sequence Length	Level
1	GCA_000732925.1	ASM73292v1	Amycolatopsis japonica	MG417-CF17	9053857	Complete
2	GCF_000732925.1	ASM73292v1	Amycolatopsis japonica	DSM 44213	9053857	Complete
3	GCA_003312875.1	ASM331287v1	Amycolatopsis albispora	WP1	9121443	Complete
4	GCA_021391495.1	ASM2139149v1	Amycolatopsis acidiphila	KCTC 39523	8183622	Complete
5	GCF_018885265.1	ASM1888526v1	Amycolatopsis aidingensis	YIM 96748	7657695	Complete
6	GCA_000454025.1	ASM45402v1	Amycolatopsis mediterranei RB	RB	10246864	Complete
7	GCA_000400635.2	ASM40063v2	Amycolatopsis keratiniphila	HCCB10007	8982090	Complete
8	GCA_024628825.1	ASM2462882v1	Amycolatopsis roodepoortensis	ZEL-1	9489959	Complete
9	GCA_000943515.2	ASM94351v2	Amycolatopsis orientalis	B-37	9490992	Complete
10	GCF_000384295.1	ASM38429v1	Amycolatopsis balhimycina FH 1894	DSM 44591	10858503	Scaffold
11	GCF_001953865.1	ASM195386v1	Amycolatopsis coloradensis	DSM 44225	9049490	Scaffold
12	GCA_001995215.1	ASM199521v1	Amycolatopsis azurea DSM 43854	DSM 43854	9217912	Scaffold
13	GCF_900115565.1	IMG-taxon 2675903051 annotated assembly	Amycolatopsis arida	CGMCC 4.5579	5955155	Scaffold
14	GCA_900110575.1	IMG-taxon 2634166333 annotated assembly	Amycolatopsis saalfeldensis	DSM 44993	9861953	Scaffold
15	GCF_000383915.1	ASM38391v1	Amycolatopsis benzoatilytica AK 16/65	AK 16/65	8704271	Scaffold
16	GCF_000519205.1	ASM51920v1	Amycolatopsis taiwanensis DSM 45107	DSM 45107	8777509	Scaffold
17	GCA_900107045.1	IMG-taxon 2675903050 annotated assembly	Amycolatopsis xylanica	CPCC 202699	9406090	Scaffold
18	GCF_003937945.1	ASM393794v1	Amycolatopsis eburnea	GLM-1	10230128	Scaffold
19	GCA_900111885.1	IMG-taxon 2667527405 annotated assembly	Amycolatopsis marina	CGMCC 4.3568	7020699	Scaffold
20	GCF_004522235.1	ASM452223v1	Amycolatopsis nivea	CFH S0261	9571567	Scaffold
21	GCF_009765355.1	ASM976535v1	Amycolatopsis anabasis	EGI 650086	10940472	Scaffold
22	GCA_021654135.1	ASM2165413v1	Amycolatopsis tucumanensis	ABO	9017186	Scaffold
23	GCF_003600245.1	ASM360024v1	Amycolatopsis panacis	YIM PH21725	6978358	Scaffold

24	GCF_002155975.1	ASM215597v1	Amycolatopsis kentuckyensis	NRRL B-24129	10183912	Scaffold
25	GCA_014654365.1	ASM1465436v1	Amycolatopsis oliviviridis	CGMCC 4.7683	9448616	Scaffold
26	GCF_014653945.1	ASM1465394v1	Amycolatopsis deserti	CGMCC 4.7677	7743013	Scaffold
27	GCA_014654095.1	ASM1465409v1	Amycolatopsis bartoniae	CGMCC 4.7679	7813348	Scaffold
28	GCF_000220945.1	ASM22094v1	Amycolatopsis mediterranei S699	S699	10236779	Complete
29	GCA_000739085.1	ASM73908v1	Amycolatopsis methanolica 239	239	7237391	Complete
30	GCF_030285665.1	ASM3028566v1	Amycolatopsis mongoliensis	4-36	10811244	Complete
31	GCA_000282715.1	ASM28271v1	Amycolatopsis mediterranei S699	S699	10246920	Complete
32	GCF_000196835.1	ASM19683v1	Amycolatopsis mediterranei U32	U32	10236715	Complete
33	GCA_023023025.1	ASM2302302v1	Amycolatopsis sp. WQ 127309	WQ 127309	11133454	Complete
34	GCF_013364075.1	ASM1336407v1	Amycolatopsis sp. Hca4	Hca4	10972864	Complete
35	GCA_030264295.1	ASM3026429v1	Amycolatopsis sp. 2-2	2-2	10665068	Complete
36	GCF_030285645.1	ASM3028564v1	Amycolatopsis sp. DG1A-15b	DG1A-15b	10407650	Complete
37	GCA_018736145.1	ASM1873614v1	Amycolatopsis sp. CA-230715	CA-230715	10375238	Complete
38	GCF_009429145.1	ASM942914v1	Amycolatopsis sp. YIM 10	YIM 10	10352908	Complete
39	GCA_023008405.1	ASM2300840v1	Amycolatopsis sp. FBCC-B4732	FBCC-B4732	10190959	Complete
40	GCF_030285625.1	ASM3028562v1	Amycolatopsis sp. 2-15	2-15	10050063	Complete
41	GCA_021903635.1	ASM2190363v1	Amycolatopsis sp. FU40	FU40	10137108	Complete
42	GCF_016889705.1	ASM1688970v1	Amycolatopsis sp. FDAARGOS 1241	FDAARGOS_1 241	9798102	Complete
43	GCA_002849735.1	ASM284973v1	Amycolatopsis sp. BJA-103	BJA-103	9488490	Complete
44	GCF_019330265.1	ASM1933026v1	Amycolatopsis sp. TNS106	TNS106	8895521	Complete
45	GCA_002796545.1	ASM279654v1	Amycolatopsis sp. AA4	AA4	9172799	Complete
46	GCF_029369745.1	ASM2936974v1	Amycolatopsis sp. QT-25	QT-25	8010145	Complete
47	GCA_026802195.1	ASM2680219v1	Amycolatopsis sp. HUAS 11-8	HUAS 11-8	7474574	Complete
48	GCF_023376025.1	ASM2337602v1	Amycolatopsis thermalba	KNN 49.3e	7285716	Complete
49	GCA_026017845.1	ASM2601784v1	Amycolatopsis mediterranei	ISP5501	10606055	Chromosome
50	GCF_900105855.1	IMG-taxon 2634166275 annotated assembly	Amycolatopsis keratiniphila	FH 1893	9472622	Chromosome
51	GCA_019468465.1	ASM1946846v1	Amycolatopsis sp. DSM 110486	DSM 110486	10984256	Chromosome
52	GCF_022479115.1	ASM2247911v1	Amycolatopsis sp. EV170708-02-1	EV170708-02-1	9660487	Chromosome

53	GCA_001558125.2	ASM155812v2	Amycolatopsis regifaucium	GY080	8283019	Scaffold
54	GCF_001613935.1	ASM161393v1	Amycolatopsis regifaucium	GY080	8273016	Scaffold
55	GCA_001953855.1	ASM195385v1	Amycolatopsis keratiniphila subsp. nogabecina	FH 1893	9399778	Scaffold
56	GCF_001620365.2	ASM162036v2	Amycolatopsis keratiniphila subsp. keratiniphila	DSM 44409	9091838	Scaffold
57	GCF_002156025.1	ASM215602v1	Amycolatopsis pretoriensis	NRRL B-24133	10112372	Scaffold
58	GCF_000473265.1	ASM47326v1	Amycolatopsis thermoflava N1165	N1165	8693468	Scaffold
59	GCF_002156005.1	ASM215600v1	Amycolatopsis lexingtonensis	NRRL B-24131	10536026	Scaffold
60	GCF_001742805.1	ASM174280v1	Amycolatopsis mediterranei	HP-130	10335541	Scaffold
61	GCA_030010455.1	ASM3001045v1	Amycolatopsis magusensis	40447	9099986	Scaffold
62	GCF_030269425.1	ASM3026942v1	Amycolatopsis taiwanensis	NBRC 103393	8717394	Scaffold
63	GCA_001905755.1	ASM190575v1	Amycolatopsis sp. CB00013	CB00013	9016033	Scaffold
64	GCF_030269325.1	ASM3026932v1	Amycolatopsis sp. NBRC 101858	NBRC 101858	11164655	Scaffold
65	GCF_021653975.1	ASM2165397v1	Amycolatopsis sp. GM8	GM8	8157730	Scaffold
66	GCA_031625235.1	ASM3162523v1	Amycolatopsis sp. 505	505	9855896	Scaffold
67	GCF_000754115.1	ASM75411v1	Amycolatopsis sp. MJM2582	MJM2582	8932977	Scaffold
68	GCA_031082405.1	ASM3108240v1	Amycolatopsis sp. A133	A133	10053543	Scaffold
69	GCF_004522315.1	ASM452231v1	Amycolatopsis sp. CFH S0740	CFH S0740	9841780	Scaffold
70	GCF_017308975.1	ASM1730897v1	Amycolatopsis sp. MtRt-6	MtRt-6	10575343	Scaffold
71	GCF_002904295.1	ASM290429v1	Amycolatopsis sp. CA-128772	CA-128772	10162752	Scaffold
72	GCF_029087445.1	ASM2908744v1	Amycolatopsis sp. La24	La24	9785700	Scaffold
73	GCA_001651785.1	ASM165178v1	Amycolatopsis sp. M39	M39	9909969	Scaffold
74	GCF_030405835.1	ASM3040583v1	Amycolatopsis sp. MEP2-6	MEP2-6	10252809	Scaffold
75	GCF_000384215.1	ASM38421v1	Amycolatopsis alba DSM 44262	DSM 44262	9811274	Scaffold
76	GCF_004522265.1	ASM452226v1	Amycolatopsis sp. CFH S0078	CFH S0078	9995835	Scaffold
